# Marine Community Metabolomes Carry Fingerprints of Phytoplankton Community Composition

**DOI:** 10.1128/mSystems.01334-20

**Published:** 2021-05-04

**Authors:** Katherine R. Heal, Bryndan P. Durham, Angela K. Boysen, Laura T. Carlson, Wei Qin, François Ribalet, Angelicque E. White, Randelle M. Bundy, E. Virginia Armbrust, Anitra E. Ingalls

**Affiliations:** a School of Oceanography, University of Washington, Seattle, Washington, USA; b Department of Biology, Genetics Institute, University of Florida, Gainesville, Florida, USA; c Department of Microbiology and Plant Biology, University of Oklahoma, Norman, Oklahoma, USA; d Department of Oceanography, University of Hawaii at Manoa, Honolulu, Hawaii, USA; e Daniel K. Inouye Center for Microbial Oceanography: Research and Education, Honolulu, Hawaii, USA; Max Planck Institute for Marine Microbiology

**Keywords:** phytoplankton, metabolomics, North Pacific, HILIC, homarine, trigonelline, gonyol, diatoms, microbial ecology, microbial loop

## Abstract

Phytoplankton transform inorganic carbon into thousands of biomolecules that represent an important pool of fixed carbon, nitrogen, and sulfur in the surface ocean. Metabolite production differs between phytoplankton, and the flux of these molecules through the microbial food web depends on compound-specific bioavailability to members of a wider microbial community. Yet relatively little is known about the diversity or concentration of metabolites within marine plankton. Here, we compare 313 polar metabolites in 21 cultured phytoplankton species and in natural planktonic communities across environmental gradients to show that bulk community metabolomes reflect the chemical composition of the phytoplankton community. We also show that groups of compounds have similar patterns across space and taxonomy, suggesting that the concentrations of these compounds in the environment are controlled by similar sources and sinks. We quantify several compounds in the surface ocean that represent substantial understudied pools of labile carbon. For example, the N-containing metabolite homarine was up to 3% of particulate carbon and is produced in high concentrations by cultured *Synechococcus*, and S-containing gonyol accumulated up to 2.5 nM in surface particles and likely originates from dinoflagellates or haptophytes. Our results show that phytoplankton composition directly shapes the carbon composition of the surface ocean. Our findings suggest that in order to access these pools of bioavailable carbon, the wider microbial community must be adapted to phytoplankton community composition.

**IMPORTANCE** Microscopic phytoplankton transform 100 million tons of inorganic carbon into thousands of different organic compounds each day. The structure of each chemical is critical to its biological and ecosystem function, yet the diversity of biomolecules produced by marine microbial communities remained mainly unexplored, especially small polar molecules which are often considered the currency of the microbial loop. Here, we explore the abundance and diversity of small biomolecules in planktonic communities across ecological gradients in the North Pacific and within 21 cultured phytoplankton species. Our work demonstrates that phytoplankton diversity is an important determinant of the chemical composition of the highly bioavailable pool of organic carbon in the ocean, and we highlight understudied yet abundant compounds in both the environment and cultured organisms. These findings add to understanding of how the chemical makeup of phytoplankton shapes marine microbial communities where the ability to sense and use biomolecules depends on the chemical structure.

## INTRODUCTION

In the ocean, the molecular makeup of organic carbon shapes its journey through the global carbon cycle. Phytoplankton fix approximately 100 million tons of carbon on a daily basis (1), roughly equivalent to half the total biomass of humans on earth (2). Each day, the microbial community respires about half of this carbon through the microbial loop (3). Approaches analyzing gene expression suggest freshly fixed small biomolecules, or metabolites, are among the most bioavailable in the surface ocean and represent a substantial conduit of carbon and energy flux. Much of the chemical complexity in phytoplankton-derived organic matter remains poorly described both qualitatively and quantitatively, particularly the highly labile portion of organic matter encompassing small polar metabolites. Here, we characterize the small molecules within particulate organic matter in natural marine microbial communities in the North Pacific and cultures of 21 phytoplankton species to show that the chemical character of the bulk carbon pool in the ocean reflects the taxonomy of the primary producers present.

Small polar metabolites can be major carbon, nutrient, and/or energy sources for heterotrophs (4, 5) and are often considered the currency of the microbial loop in the ocean. Beyond this, they can maintain phytoplankton-bacterial interactions (6, 7), serve as micronutrients (8–10), manage redox stress (11), fuel nitrogen fixation (12), act as chemical defenses (13, 14), and more. The comprehensive analysis of the metabolites in a system (metabolomics) is a nascent field and analytically challenging in environmental settings (15–17). Metabolomic studies are being used to investigate physiological changes in marine organisms under laboratory conditions (4, 18–22), though the same techniques have only recently been applied to whole communities in natural environments (22). Existing community marine metabolomic studies have employed targeted approaches in which the compounds detected are chosen by the analyst (5, 12, 21, 23, 24) or the analytical techniques employed preclude the observation of small, highly polar compounds (25, 26).

The chemical makeup of small polar compounds in freshly fixed organic matter influences the flux of carbon and energy through the microbial loop in the surface ocean. Here, we determine the metabolite pools in natural marine communities across space to explore the distributions of both known and unknown compounds. We compare our field observations to metabolomes of cultured marine primary producers from a broad taxonomic range and show how primary producers play an active role in shaping the chemical environment of the surface ocean. Finally, we highlight small polar compounds that may serve as potentially significant conduits of energy and nutrients in marine systems.

## RESULTS AND DISCUSSION

### Patterns of metabolites across space and taxonomy.

Using an established untargeted metabolomics approach, we obtained a list of mass features (liquid chromatography-mass spectrometry [LC-MS]-derived peaks of a particular mass and time) from marine particles. We dereplicated these mass features for adducts and isotopes, performed extensive quality control and blank comparisons, and visually inspected the resulting mass features. Through this process, we obtained a curated list of mass features, which we refer to as metabolites throughout the paper. Before statistical analyses, peak areas of each metabolite were normalized for instrument-derived obscuring variability and volume sampled as noted in Materials and Methods. To examine abundance patterns of compounds, we normalized peak areas to the total observed peak area in each sample set. See Materials and Methods for details on each step.

**FIG 1:**
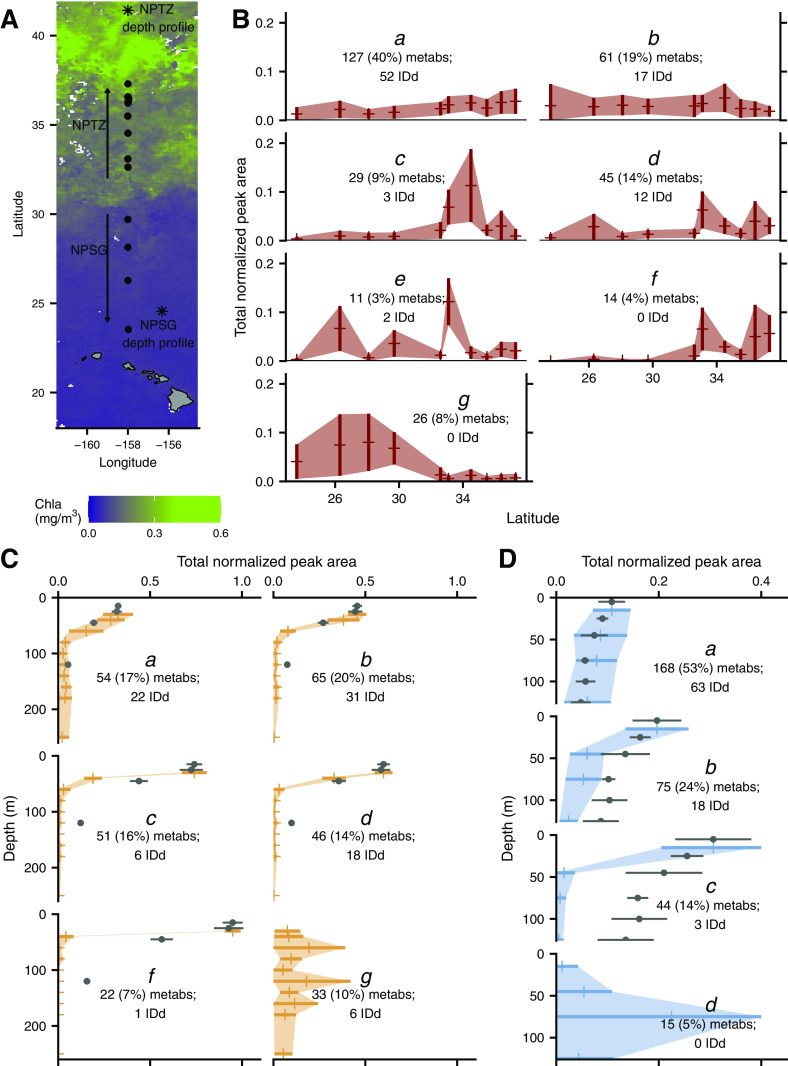
Sampling location and metabolite patterns. Map of sample locations of transect samples (dot) and depth profiles (asterisks). Map is overlaid with satellite-derived (MODIS-Aqua) chlorophyll at 8-day, 9-km resolution over the time period of the transect sampling (A). Patterns of total normalized metabolite concentrations found in each environmental data set grouped into modes as a result of *k*-medoids clustering, plotted as 1 standard deviation around the mean (*a* to *g* in panel B, meridional transect; *a* to *d*, *f*, and *g* in panel C, North Pacific Transition Zone [NPTZ] depth profile; *a* to *d* in panel D, North Pacific Subtropical Gyre [NPSG] depth profile). We have excluded modes with fewer than 10 compounds in each data set. Maximum normalized bulk PC is plotted over depth profiles, with surface PC concentration plotted to match surface total normalized metabolite peak area in order to compare the shape of attenuation (excluded in modes that do not attenuate with depth). Gray dots with error bars (standard deviation, often smaller than markers). Number of metabolites (metabs) and percentage of metabolites assigned to each cluster are noted, as well as the number of compounds identified (IDd) in each cluster. Full results are presented in Table S5 with cluster assignments in Table S6, both at https://doi.org/10.5061/dryad.brv15dv8s.

We explored the patterns of metabolite abundances in three sample sets of marine particles in the North Pacific Ocean: one surface meridional transect and two depth profiles (Fig. 1A; see also Table S1 in the supplemental material). In the transect sample set, seven general patterns emerged across latitude using a *k*-medoids clustering approach of 313 metabolites (Fig. 1B). The most common pattern (40% of compounds) showed a modest increase in concentration with latitude (mode *a* in Fig. 1B). This is likely related to the general increase in biomass with latitude (Fig. S1A and B and as seen in the increase of chlorophyll in Fig. 1A). Many compounds (30%) had their highest concentration in samples from 33 or 34°N (Fig. 1B, modes *c* to *f*). About 19% of metabolites did not have a clear pattern with latitude (Fig. 1B, mode *b*), while 8% of the compounds were generally more abundant in the southern samples than the northern samples (Fig. 1B, mode *g*).

10.1128/mSystems.01334-20.1 FIG S1 Latitudinal trends in particulate carbon and fucoxanthin. (A) Particulate carbon over the April 2016 transect. (B) Total quantifiable particulate metabolites over the April 2016 transect. (C to E) Particulate carbon estimated by particular populations of phytoplankton as observed via underway flow cytometry (SeaFlow): *Synechococcus* (C), *Prochlorococcus* (D), and picoeukaryotes (E). (F) Concentration of the pigment fucoxanthin over latitude in transect samples. Phytoplankton carbon (C, D, and E) is binned every 0.5 degree of latitude, and total PC (A) is binned every 1 degree of latitude. Gray shading shows standard deviation. All particulate carbon is in micromole C liter^−1^. This is the northbound transect only. Download FIG S1, PDF file, 0.02 MB. Copyright © 2021 Heal et al. 2021 Heal et al. https://creativecommons.org/licenses/by/4.0/ This content is distributed under the terms of the Creative Commons Attribution 4.0 International license.

10.1128/mSystems.01334-20.8 TABLE S1 Summary of samples collected and analyzed in this study. Download Table S1, PDF file, 0.4 MB. Copyright © 2021 Heal et al. 2021 Heal et al. https://creativecommons.org/licenses/by/4.0/ This content is distributed under the terms of the Creative Commons Attribution 4.0 International license.

In a nonmetric multidimensional scaling (NMDS) analysis where each metabolite was treated with equal weight, there was a distinct shift in metabolite patterns in the samples on either side of approximately 30°N (Fig. S2A, analysis of similarity [ANOSIM] stat = 0.316, *P* = 0.005). This corresponds well with the southern boundary of the North Pacific Transition Zone (NPTZ), a well described oceanographic feature which extends from Japan to North America and arises from large-scale ocean circulation (27, 28). The northern and southern edges of this transition zone comprise rapid changes in thermohaline structure and biological species composition (27, 29, 30). We saw a similar stark transition within the metabolite pools that reiterate the transition from the warm, oligotrophic North Pacific Subtropical Gyre (NPSG) into the colder, more nutrient-replete North Pacific Transition Zone (NPTZ) where chlorophyll concentrations, *Synechococcus*, and picoeukaryote assemblages flourish (Fig. 1 and Fig. S1C to E; see Table S2 for oceanographic conditions). Interestingly, the difference between metabolite profiles within the northern samples encompasses a much wider range in multivariate space, even within samples collected at the same time and location (biological replicates, Fig. S2A). This suggests the NPTZ is more heterogeneous in its metabolite profiles than the NPSG and is supported by the observed high variability in particulate carbon (PC) at northern sampling sites (Fig. S1A).

10.1128/mSystems.01334-20.2 FIG S2 Nonmetric multidimensional scaling comparison of metabolite composition on the KOK1606 transect (A) and organisms (B), *P* < 0.01 by Monte Carlo permutation for both. (A) Colors are based on latitude of samples; biological replicates shown connected, with samples from NPSG in circles and NPTZ in triangles. (B) Colors are based on broad taxonomic groups; biological replicates are shown connected. Both plots are based on Euclidean distance between standardized adjusted peak areas. Download FIG S2, PDF file, 0.01 MB. Copyright © 2021 Heal et al. 2021 Heal et al. https://creativecommons.org/licenses/by/4.0/ This content is distributed under the terms of the Creative Commons Attribution 4.0 International license.

10.1128/mSystems.01334-20.9 TABLE S2 Summary of physical and chemical parameters on April 2016 cruise. Reprinted from reference 28 with permission of the publisher. Download Table S2, PDF file, 0.1 MB. Copyright © 2021 Heal et al. 2021 Heal et al. https://creativecommons.org/licenses/by/4.0/ This content is distributed under the terms of the Creative Commons Attribution 4.0 International license.

We performed the same clustering technique (*k*-medoids) on the same metabolites (when observed) within two depth profiles: one from the NPSG and one from the NPTZ (Fig. 1A for locations). Most of the metabolite concentrations decreased with depth, again corresponding with a decrease of PC (Fig. 1C, modes *a* to *f*, and Fig. 1D, modes *a* to *c*). The extent of decrease in concentrations varied among metabolites, exemplified in the NPTZ depth profile by comparing the sharply attenuating mode *c* to the gentler attenuation of mode *a* (Fig. 1C). Modes *a* in both NPTZ and NPSG depth profiles follow the PC pattern closely, but the other modes do not follow the same trend as bulk PC with depth (Fig. 1C and D; data in Table S9 at https://doi.org/10.5061/dryad.brv15dv8s). A minority of compounds in both of these depth profiles either had a subsurface maximum or had no clear relationship with depth (Fig. 1C, mode *g*, and Fig. 1D, mode *d*).

**FIG 2:**
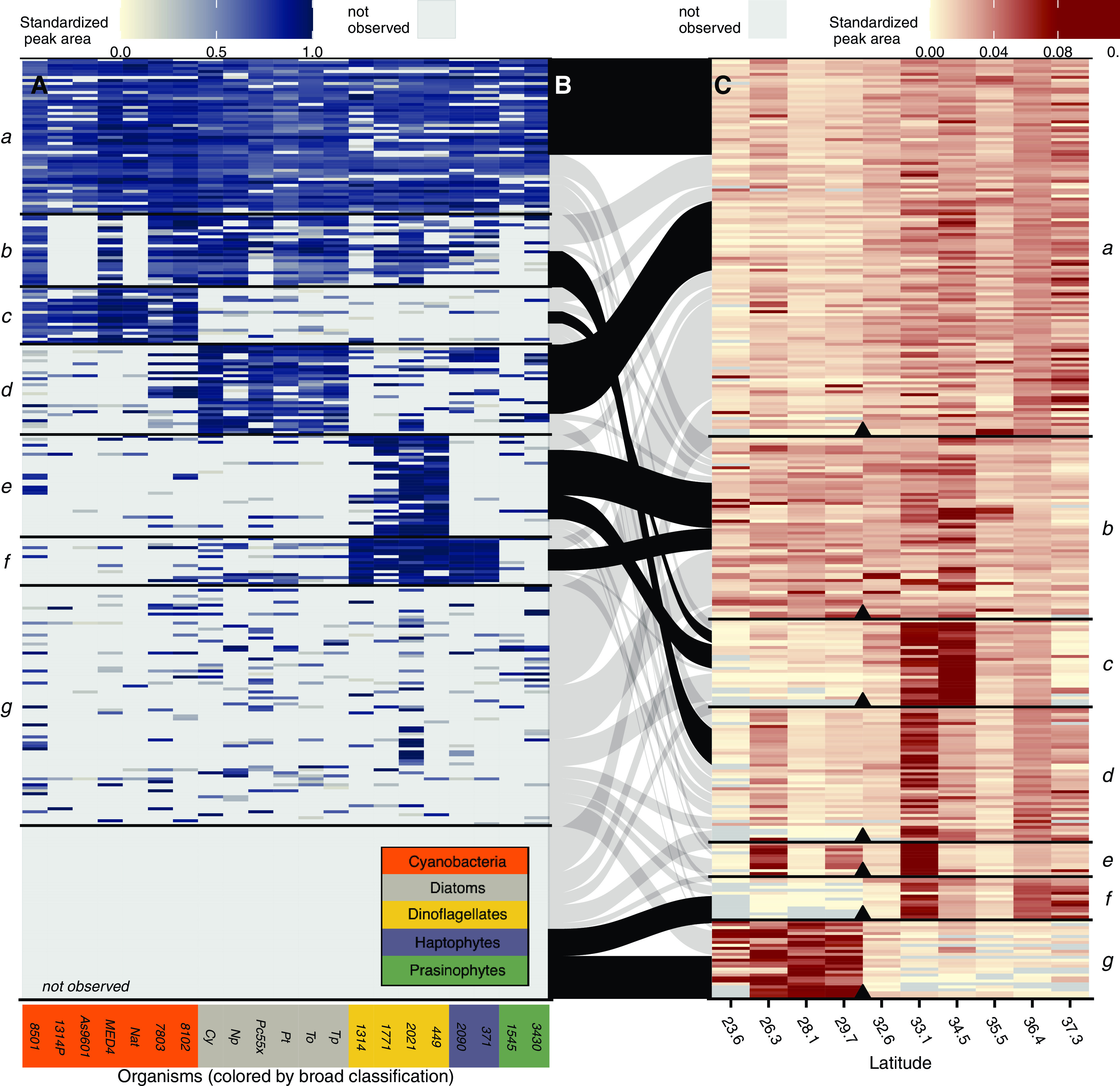
Relative abundance patterns in environmental and culture metabolites. Each row is a metabolite—either identified or unknown. Left (A) is the standardized peak areas for metabolites in the culture data sets. Right (C) is the relative abundance between samples along the meridional transect. Tile panels are grouped separately using a *k*-medoids clustering and reordered within each mode for visual clarity. The middle panel (B) shows which metabolites are shared between the culture and environmental *k*-medoids-derived modes, with overenriched connections between modes shown in black (*P* < 0.05 by bootstrap test) and remaining non-statistically significant connections shown in gray. Organisms are colored by broad taxonomic classification as shown in inset (orange = cyanobacteria, gray = diatoms, yellow = dinoflagellates, purple = haptophytes, green = prasinophytes).

Using the 313 metabolites from the transect sample set as a template, we searched for the same compounds within metabolomes of 21 species of axenic phytoplankton grown under controlled conditions and analyzed on the same instrumental setup (5) (Table S3; also see Table S7 at https://doi.org/10.5061/dryad.brv15dv8s). Phytoplankton are the primary source of fixed carbon to the surface ocean, and our cultures were grown under conditions that support autotrophic growth so that we could interrogate the metabolite pools these organisms produce *de novo* from inorganic components. The cultures explored here encompass a wide taxonomic range from picocyanobacteria that dominate much of our transect (Fig. S1) to members of ubiquitous eukaryotic phytoplankton lineages like diatoms and coccolithophores. The taxonomic groups were recapitulated after a multivariate analysis of the metabolites across this data set in a semiquantitative manner, using both NMDS (Fig. S2B) and *k*-medoids clustering (Fig. 2A).

10.1128/mSystems.01334-20.10 TABLE S3 Summary of cultured organisms analyzed in this study. Download Table S3, PDF file, 0.4 MB. Copyright © 2021 Heal et al. 2021 Heal et al. https://creativecommons.org/licenses/by/4.0/ This content is distributed under the terms of the Creative Commons Attribution 4.0 International license.

Overall, we saw that 17% (52) of the 313 metabolites were present in most of the cultured organisms with 44 of the metabolites within this mode observed in over 80% of the phytoplankton species (mode *a* in Fig. 2A). This suggests a set of compounds observable in most phytoplankton when analyzed under our analytical conditions. We were able to identify most of these compounds (33/52, 64%), which include many amino acids, primary metabolites, and nucleic acids (see Tables S5 and S6 at https://doi.org/10.5061/dryad.brv15dv8s). The remaining 36% of compounds within this mode could not be identified, demonstrating that even the compounds critical to the physiology and biochemistry of a broad swath of marine primary producers remain elusive. Another 39% (123) of metabolites were seen primarily in subsets of organisms, separating into five modes (modes *b* to *f* in Fig. 2A). Finally, about 44% of the 313 metabolites were either rarely or never observed in our cultures (mode *g* and “not observed” in Fig. 2A).

The patterns of metabolites across the cultures suggest suites of compounds that are closely associated with taxonomic groups of organisms. Several identified metabolites in these groups corroborate previous work showing that certain types of organisms produce high concentrations of particular small molecules. For instance, 2,3-dihydroxypropane-1-sulfonate (DHPS) and isethionate are within the mode of metabolites associated with diatoms (mode *d*) (5, 7), taurine is associated with dinoflagellates and haptophytes (mode *f*) (5), and glucosylglycerol is associated with cyanobacteria (31) (mode *c*, Fig. 2A; see also Table S6 at https://doi.org/10.5061/dryad.brv15dv8s). Most of the taxon-associated metabolites (72% of metabolites in modes *b* to *f* in Fig. 2A) are still unidentified and offer possible future taxon-specific biomarkers in the polar organic carbon pools.

### Primary producers leave a metabolite signature in the environment.

Compounds with similar patterns across these data sets would suggest shared sources and sinks. To assess this, we tested whether each *k*-medoids-derived mode (within each sample set) was enriched in metabolites from a given mode from a different sample set, beyond what would be expected with a random assignment (assessed by a Monte Carlo-based bootstrapping approach, *P* value < 0.05). For example, of the 52 metabolites that were observed in most of our cultured phytoplankton (mode *a* in Fig. 2A), there was a robust enrichment of compounds from the meridional transect mode *a* (in Fig. 2C; general increase with latitude, *P* < 0.01; Fig. 2B). This may reflect a general increase in phytoplankton biomass with latitude as supported by the increase in PC and chlorophyll (Fig. S1A and Fig. 1A).

**FIG 3:**
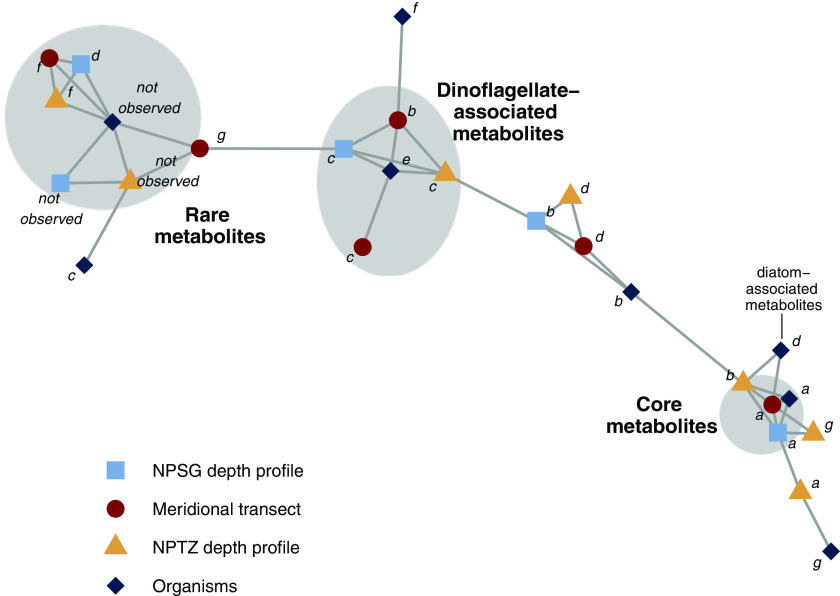
Network visualization of significant overlap between data sets shown in a network visualization. Each mode in each data set is depicted as a node (colored and shaped by data set, labeled as in Fig. 1 and 2). Metaclusters are highlighted in gray and labeled as described in the text. Edges are connections between modes that are overenriched in the same compounds (*P* < 0.05). Compound assignments to each mode and metacluster (if applicable) are found in Table S6 at https://doi.org/10.5061/dryad.brv15dv8s.

We capitalized on our results to search for enrichment between each mode in each sample set and visualized enriched connections among all sample sets in a network analysis where each connection is a statistically significant enrichment between two modes (*P* < 0.05 by Monte Carlo permutation, Fig. 3). This analysis revealed a few metaclusters, or groups of compounds that have similar patterns as each other across different spatial and taxonomic ranges (Fig. 3). These metaclusters suggest compounds have similar sources across taxonomy that persist across both latitudinal and depth gradients in the environment. Taxonomy here is represented by the modes derived from the culture data set, presented in Fig. 2A, and corresponding modes are depicted as dark blue markers in Fig. 3. Building on the observation of overenrichment of metabolites between transect data set mode *a* and culture data set mode *a*, we also saw that these modes share metabolites well beyond random assignment with the modes of metabolites in both depth profiles that attenuated in close proportion to PC with depth (modes *a* and *b* in NPTZ depth profile, mode *a* in NPSG depth profile, *P* < 0.05, Fig. 3). Identified compounds within this “core metabolome” metacluster included the amino acid glutamic acid, the nucleoside adenosine, the amino acid precursor homoserine, and several other primary metabolites (see Table S6 at https://doi.org/10.5061/dryad.brv15dv8s).

Beyond the “core metabolome,” 77% of the 30 compounds associated tightly with diatoms (mode *d* in Fig. 2A) were also overrepresented within the group of compounds with a general increase with latitude (*P* < 0.01, Fig. 2B). This pattern corresponded with an increase in fucoxanthin, a diatom biomarker observed to increase with latitude in a separate analysis from the same sampling period (Fig. S1F). The diatom-associated metabolites were also overrepresented in the medium-attenuating metabolites from the NPTZ depth profiles (mode *b* in Fig. 1C and Fig. 3). DHPS (a probable osmolyte produced in high concentrations by diatoms [5, 21]) and glycerophosphocholine (a headgroup of phosphatidylcholine lipids commonly produced by eukaryotic phytoplankton in marine systems [32]) sat within this pattern space as did 11 other unidentified compounds (see Table S6 at https://doi.org/10.5061/dryad.brv15dv8s).

Surprisingly, compounds tightly associated with dinoflagellates (mode *e* in Fig. 2A) showed a significant overrepresentation with an environmental distribution showing a distinct increase in concentration at 34.5°N (Fig. 2). Metabolites displaying these patterns were overrepresented in the sharply attenuating modes in the two depth profiles (dinoflagellate-associated metacluster in Fig. 3), in contrast to the metabolites found associated with diatoms. None of the compounds that reside in this interaction space could be identified, leaving room for future work to identify and leverage these compounds as possible biomarkers for dinoflagellates that are easily observable in the environment.

The group of compounds observed in our environmental samples but not observed in our culture data set (“not observed” mode in Fig. 2A) were overrepresented in compounds that were more abundant in the NPSG than the NPTZ in the transect (mode *g* in Fig. 2C) or increased with depth in the two depth profiles (rare metabolites metacluster in Fig. 3). We analyzed phytoplankton only in our initial analysis; therefore, it is likely that a subset of these compounds were produced by organisms we did not survey. For instance, the compound β-glutamic acid was found to be more abundant at depth than in the surface waters in both of our depth profiles, in contrast to the majority of compounds observed (Fig. S4 inset) and was absent from our phytoplankton cultures (see Table S5 at https://doi.org/10.5061/dryad.brv15dv8s). β-Glutamic acid is a major osmolyte in methanogenic archaea (33, 34), prompting us to search for this compound in Nitrosopumilus maritimus strain SCM1, a model species of Marine Group I *Thaumarchaeota* that are abundant in the ocean’s subsurface (35). We grew *N. maritimus*, analyzed its metabolome, and found β-glutamic acid as the most abundant identified metabolite, present at an intracellular concentration of 730 mM (see Table S6 at https://doi.org/10.5061/dryad.brv15dv8s).

**FIG 4:**
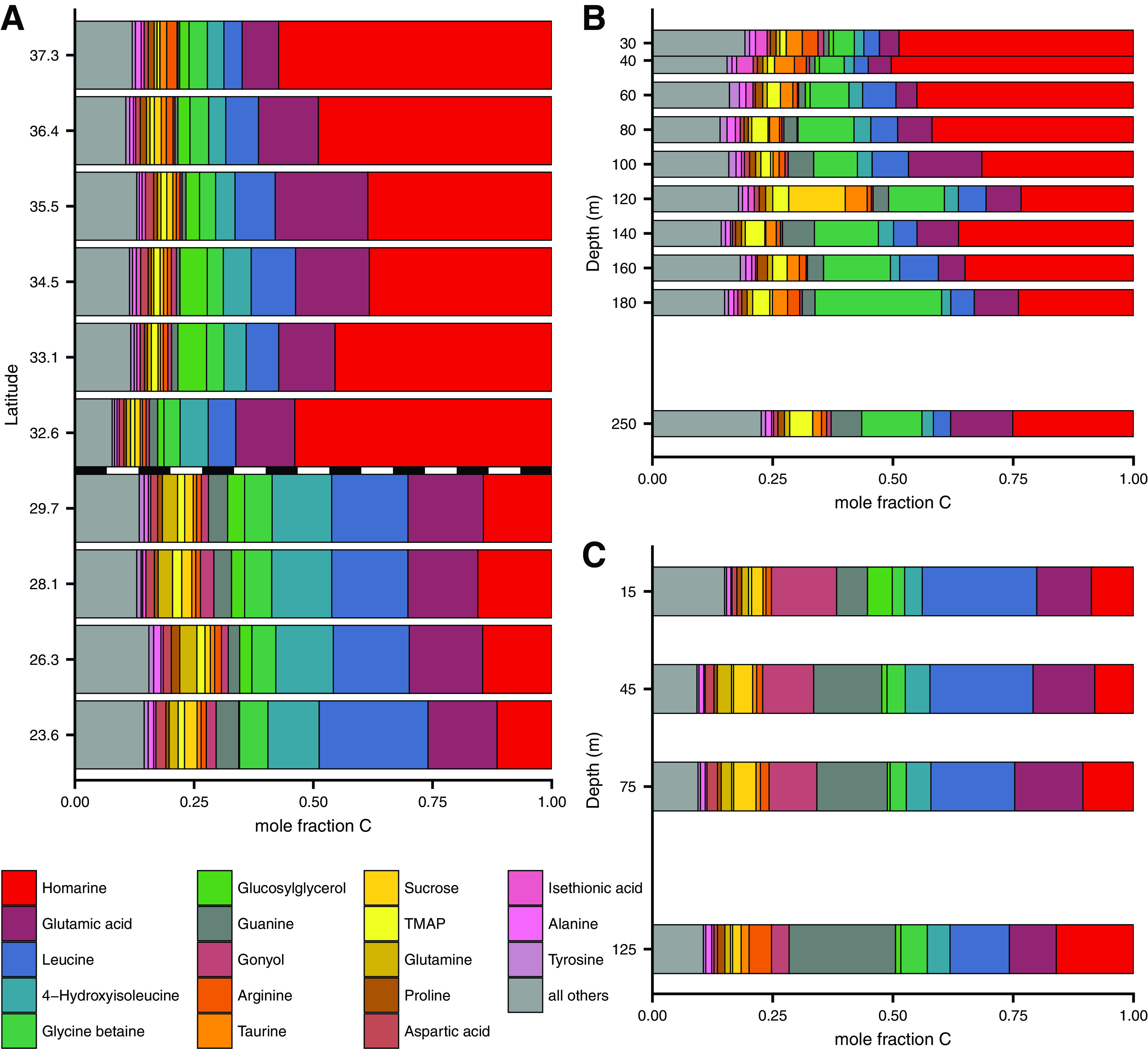
The 18 most abundant metabolites in environmental samples, presented as mole fraction of carbon of total identified metabolites. (A) Meridional transect, with transition between NPSG and NPTZ shown as dashed line. (B and C) Depth profile from NPTZ (B) and NPSG (C). Locations of samples are shown in Fig. 1. These same data are presented as nmol C per liter in Fig. S4; full results are in Table S10 at https://doi.org/10.5061/dryad.brv15dv8s. Note that the *y* axis in panel A is not in latitudinal space for easier viewing and that we have excluded DMSP from this analysis. TMAP = trimethylammonium propionate.

10.1128/mSystems.01334-20.4 FIG S4 The 18 most abundant metabolites in environmental samples, presented as nanomoles carbon per liter. (A) Meridional transect, with transition between NPSG and NPTZ shown as dotted line. (B and C) Depth profile from NPTZ (B) and NPSG (C). Locations of samples are shown in Fig. 1. These same data are presented as carbon mole fraction in Fig. 4; full results are in Table S9 at https://doi.org/10.5061/dryad.brv15dv8s. Inset: β-glutamic acid (closed circles) and arsenobetaine (open circles) depth profiles from the NPTZ. Note the different scales. Download FIG S4, PDF file, 0.01 MB. Copyright © 2021 Heal et al. 2021 Heal et al. https://creativecommons.org/licenses/by/4.0/ This content is distributed under the terms of the Creative Commons Attribution 4.0 International license.

It is likely that some compounds in this group were not made “freshly” by primary producers like phytoplankton or ammonia-oxidizing archaea but were rather a signature of reworked particulate matter. For example, the compound arsenobetaine, which we detected in all of our environmental samples, similarly to β-glutamic acid, generally increased with depth in the depth profiles (Fig. S4 inset). This compound is a by-product of heterotrophic degradation of phytoplankton-produced arsenometabolites (36) and would therefore necessitate a co-culture in order to be observed in a laboratory setting (as well as a growth medium with arsenic). Finally, it is likely that the cultures explored here were not producing all the compounds they are genetically able to produce—in previous laboratory experiments certain metabolites accumulate in cultures under specific environmental conditions and are not detectable under other environmental conditions (20, 21). If the production of certain compounds is variable or at rates below detection, we may not have seen them on our culture data.

### Metabolites as a quantitative component of the bulk carbon pool.

We obtained absolute concentrations of the identified compounds to better understand the quantitative importance of these different metabolites within the particulate carbon landscape. The combined concentration of the identified metabolites (85 of the 313 total) ranged from 68 to 234 nM particulate carbon in the surface transect samples (Fig. S1B and Fig. S4; see Table S9 at https://doi.org/10.5061/dryad.brv15dv8s). This corresponds to 2.9% (±1.0%) to 5.2% (±1.4%) of the particulate carbon pool and 2.6% (±1.0%) to 8.2% (±2.4%) of the particulate nitrogen pool across this transect (see Table S9 at https://doi.org/10.5061/dryad.brv15dv8s). There was no clear pattern in the percentage of particulate carbon or nitrogen characterized by the quantifiable metabolites with latitude; this is likely confounded by the high variability in the particulate carbon and nitrogen measurements and the low geographical resolution of the metabolite sampling. In the NPTZ depth profile, we quantified 17 to 966 nM particulate carbon in the metabolite pool, corresponding to a rough estimate of 10% of the particulate carbon and nitrogen pools in the surface sample (see Table S9 at https://doi.org/10.5061/dryad.brv15dv8s). In the NPSG profile, we quantified approximately 3.7% of the total carbon pool in the surface sample (see Table S9 at https://doi.org/10.5061/dryad.brv15dv8s). The concentration of surface particulate metabolites was approximately two times higher than what we observed a year later in the NPSG (during the transect sampling; see Table S9 at https://doi.org/10.5061/dryad.brv15dv8s), likely due to the fact that the NPSG depth profile was sampled within an anticyclonic eddy with high surface primary productivity and particulate carbon (37).

Quantitatively, the environmental metabolite pools were dominated by a few abundant compounds, similar to previous work (12, 24). There were obvious differences in metabolite composition between the three environmental samplings (Fig. 4). For example, on a molar basis, glycine betaine (GBT) contributed to up to 17% of the quantified metabolite pool in samples below 125 m in the NPTZ, substantially more (on a mole fraction basis) than the other data sets. In contrast, the NPSG depth profile had high contributions from gonyol in the surface and guanine at depth.

**FIG 5:**
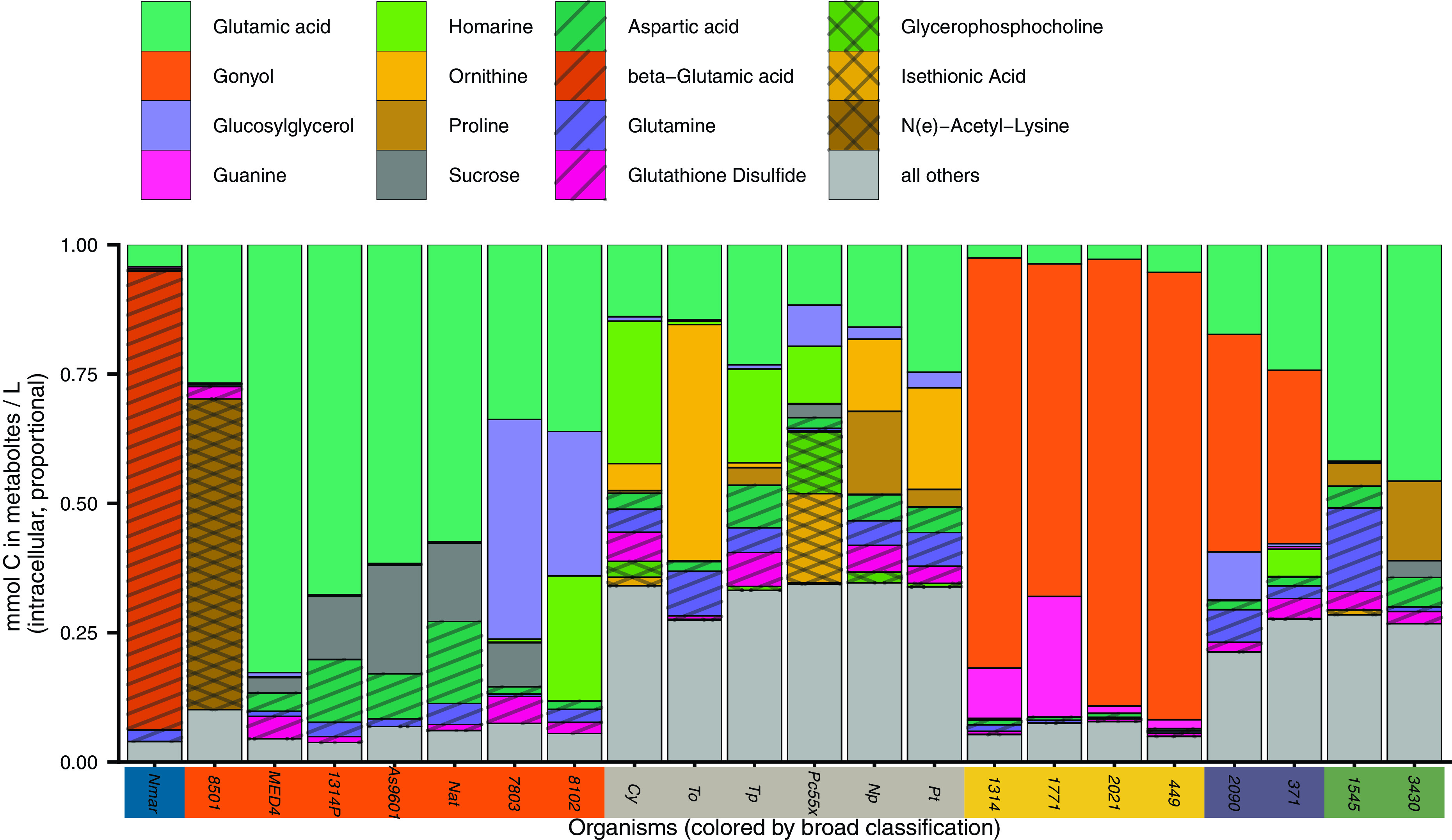
Identified metabolites in culture samples, presented as mole fraction of carbon of total identified metabolites, with at least the two most abundant compounds highlighted; full results are in Table S8 at https://doi.org/10.5061/dryad.brv15dv8s. Organisms are colored by broad taxonomic classification (as in Fig. 2; blue = archaea, orange = cyanobacteria, gray = diatoms, yellow = dinoflagellates, purple = haptophytes, green = prasinophytes), and the full description of cultured organisms is found in Table S3 in the supplemental material and Table S7, with full data in Table S8 (Tables S7 and S8 at https://doi.org/10.5061/dryad.brv15dv8s). We have excluded DMSP from this analysis since we cannot accurately quantify it using our methodology. Patterns are for added clarity with differentiating compounds.

We quantified the same molecules in the 21 species of phytoplankton and one species of *Thaumarchaeota* (Fig. 5, Fig. S5, and Table S3). Most of the abundant compounds in the environment were also found in high abundance in at least some of our cultures, though many of the most abundant compounds were not ubiquitously observed across the cultures (e.g., glycine betaine and sucrose; Fig. S5). We estimated the contribution of each metabolite to the carbon pool within each organism and compared this value to the surface samples of particulate metabolites in the field (Fig. S3). This comparison yielded a consistency suggesting most of the surface particles contain compounds that have not been heavily reworked, corroborating previous work looking at macromolecule pools (38), particularly in the compounds found within the “core metabolome” metacluster in Fig. 3 (Fig. S3). Comparing our environmental data sets to the culture data sets highlights compounds that were overrepresented in either the culture data sets or the environmental data set in a quantitative sense (Fig. S5). For example, common compounds guanine and creatine and less well studied compounds like isethionic acid and dimethylsulfonioacetate (DMS-Ac) were all higher on a per-carbon basis in the environment than in any of our cultures.

10.1128/mSystems.01334-20.3 FIG S3 All identified and quantified compounds. Each compound is shown as a dot with error bars, representing median observation and range of observations, respectively (excluding instances where we did not observe the compound), with 1:1 line plotted. Compounds that were not detected in phytoplankton are shown as open circles (x value is arbitrary). Compounds assigned to the “core” metacluster in Fig. 3 are plotted in purple. Compounds are highlighted when median observations are 10 times higher in either the environmental sample set or the culture sample set (in carbon space). These values are also reported in Table S10 at https://doi.org/10.5061/dryad.brv15dv8s. Note that both axes are in log_10_ space. Download FIG S3, PDF file, 0.01 MB. Copyright © 2021 Heal et al. 2021 Heal et al. https://creativecommons.org/licenses/by/4.0/ This content is distributed under the terms of the Creative Commons Attribution 4.0 International license.

10.1128/mSystems.01334-20.5 FIG S5 All identified compounds, with average and standard deviation (box and whiskers, respectively) of concentrations in surface seawater particles or in culture samples (excluding instances where we did not observe the compound). Right-hand tiled panels show the fraction of samples (environmental samples on the left, culture samples on the right) in which we observed these compounds. Note that *x* axis is on a log scale. Full results are found in Table S10 at https://doi.org/10.5061/dryad.brv15dv8s. Download FIG S5, PDF file, 0.03 MB. Copyright © 2021 Heal et al. 2021 Heal et al. https://creativecommons.org/licenses/by/4.0/ This content is distributed under the terms of the Creative Commons Attribution 4.0 International license.

### Homarine, an understudied metabolite of high abundance.

**FIG 6:**
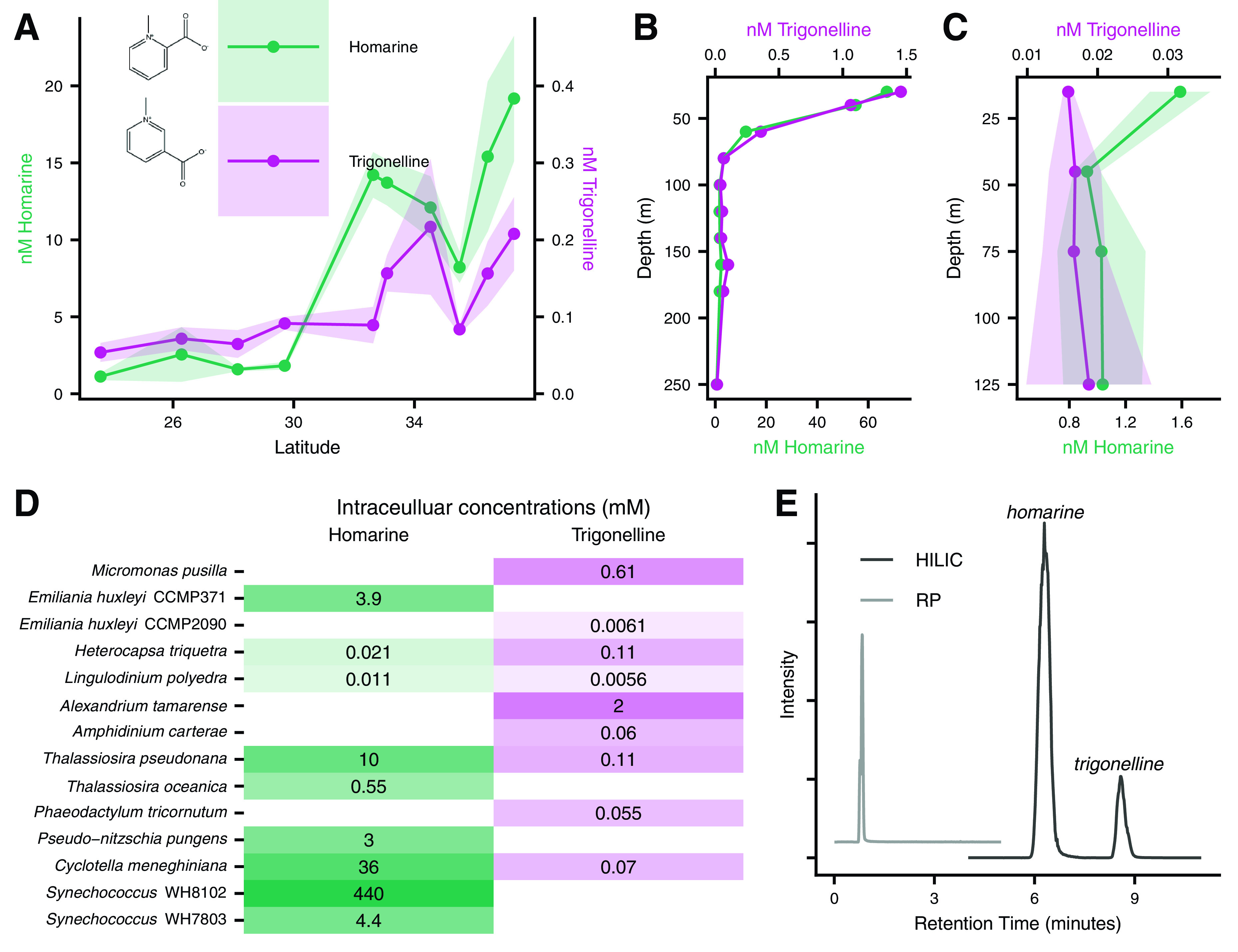
(A to C) Homarine and trigonelline spatial patterns in meridional transect (A), NPTZ depth profile (B), and NPSG depth profile (C). (D) Intracellular concentrations of homarine and trigonelline in relevant organisms. (E) Chromatograms show the separation under HILIC but not RP. Note the different scales for trigonelline and homarine (A to C) and depth (B and C).

The metabolite homarine (*N*-methylpicolinic acid) was present at 0.6 to 67 nM in marine particles, represented up to 3% of the total PC pool in our transect samples, and was the most abundant compound measured in our data sets (Fig. 4 and 6 and Fig. S5; see Table S10 at https://doi.org/10.5061/dryad.brv15dv8s). We found these concentrations surprising both in their absolute abundance and compared to other more commonly studied polar metabolites known to accumulate in marine phytoplankton. For example, other studies have shown that homarine in marine particles is less abundant than the compatible solute glycine betaine (GBT) (12, 39), contrasting with our findings. Both homarine and GBT are zwitterionic nitrogenous betaines that likely serve (at least in part) as compatible solutes. We also detected trigonelline (*N*-methylnicotinic acid), an isomer of homarine, albeit at much lower concentrations (1 to 300 pM in transect samples; Fig. 6 and Fig. S5; see Table S10 at https://doi.org/10.5061/dryad.brv15dv8s). To our knowledge, trigonelline has not been previously detected in any marine samples, though it has been highlighted as an important component of labile carbon in terrestrial ecosystems due to its accumulation in higher plants (40).

In our cultured isolates, we detected homarine in both *Synechococcus* strains (intracellular concentration up to 400 mM), four of six surveyed diatoms (0.5 to 57 mM), and one strain of Emiliania huxleyi (a haptophyte, at 3.8 mM; Fig. 6D; see also Table S8 at https://doi.org/10.5061/dryad.brv15dv8s). Homarine has been observed in diatoms and *E. huxleyi* in previous studies (20, 41–45) but has not been associated with the ubiquitous marine cyanobacterium *Synechococcus*. We estimated that homarine was 4.8% of the particulate carbon within *Synechococcus* strain WH8102. *Synechococcus* has been estimated to contribute 10 to 20% of global ocean net primary production at approximately 8 Gt C per year (46); by extrapolation this suggests up to 0.5 to 1% global ocean net primary production could be attributed to *Synechococcus* through this one molecule, with potential for more production from diatoms. A caveat to this calculation is that homarine production is not quantitatively consistent among different strains of *Synechococcus*; *Synechococcus* WH7803 produced nearly 100 times less homarine (4 to 5 mM) under the same growing conditions (Fig. 6; see also Table S8 at https://doi.org/10.5061/dryad.brv15dv8s). This estimation is a first pass with the limited data at hand, and the sizable standing stock of homarine in our northern samples (about 2% of the PC) far exceeds what we would expect from the observed *Synechococcus* standing stock, which contributes less than 10% of the total PC pool (Fig. S1). Homarine had a clear attenuation with depth in both of our depth profiles (within mode *b* in the NPTZ and mode *a* in the NPSG in Fig. 1, shown in detail in Fig. 6B and C). All together, these data support active production and cycling of this compound in the surface ocean that has been previously unnoticed.

Homarine showed a clear spatial pattern along our transect with a nearly 10-times-higher abundance in the NPTZ (average 14.3 nM) than the NPSG (average 1.85 nM) (Fig. 6A), which we hypothesize is a result of the changing phytoplankton community and increasing prevalence of *Synechococcus* around 32°N (Fig. S1) and diatoms further north (indicated by increasing fucoxanthin around 34°N, Fig. S1F). Since *Synechococcus* standing stock cannot explain the observed homarine concentrations, we hypothesize that this compound may transfer to and accumulate in organisms beyond *Synechococcus*, which has been observed for osmolytes in other systems (45). Trigonelline followed a similar pattern along the transect, but with a less pronounced increase in concentration from the NPSG (average 0.07 nM) to the NPTZ (average 0.14 nM) that was shifted more northward (Fig. 6A). Homarine decreased sharply with depth in both depth profiles while trigonelline did not show appreciable attenuation in the NPSG profile (Fig. 6B and C).

Biochemically, the sources and sinks for homarine and trigonelline are likely distinct. Trigonelline is produced from nicotinic acid (47), while homarine is decarboxylated from quinolinic acid, which is produced from tryptophan (48), though the exact enzyme that performs the decarboxylation has not been characterized. The first step of bacterial trigonelline degradation is the opening of the aromatic ring by the TgnA/TgnB oxygenase system (40). This enzymatic machinery is unlikely to operate on homarine due to steric hindrance in the ring-opening step. Supporting the differential catabolism of homarine and trigonelline, we saw that the model marine heterotrophic bacterium Ruegeria pomeroyi DSS-3 was not able to grow on homarine as effectively as trigonelline (Fig. S6). Without characterized biosynthetic or degradation pathways for homarine, it is not surprising that this metabolite has not been identified as an important component of the labile organic carbon and nitrogen pools using gene-based techniques. Our spatial patterns and divergent observations of these compounds in our cultured organisms (Fig. 6D) support distinct biological sources for these structurally similar compounds, demonstrating the intricate networks that exist in microbial communities rooted in the substrate-matched metabolisms.

10.1128/mSystems.01334-20.6 FIG S6 Growth curves of Ruegeria pomeroyi DSS-3 with three different carbon sources, acetate (positive control), homarine, and trigonelline, and no additional carbon (negative control). Note that the initial growth of the negative control is due to carryover of carbon from the inoculum, which had acetate as the carbon source. The same total carbon was added to each treatment. Download FIG S6, PDF file, 0.01 MB. Copyright © 2021 Heal et al. 2021 Heal et al. https://creativecommons.org/licenses/by/4.0/ This content is distributed under the terms of the Creative Commons Attribution 4.0 International license.

Our observations of trigonelline and homarine were possible because of the chromatography methodology we employed (hydrophilic interaction liquid chromatography [HILIC])—the compounds would not be resolved in time in more commonly employed reversed-phase (RP) chromatography (24, 26) due to their high polarity and same empirical formula (and therefore exact mass, Fig. 6E). This is also true for many sets of isomers of known compounds (e.g., sarcosine, β-alanine, and alanine; homoserine and threonine; β-glutamic acid and glutamic acid) as well as unknowns (e.g., inosine and another unidentified metabolite with the same *m/z*; two metabolites with an *m/z* of 236.1492; see Table S5 at https://doi.org/10.5061/dryad.brv15dv8s). We bring attention to this detail to highlight the power of incorporating cutting-edge analytical capabilities to study microbial ecology—without HILIC, we would not have been able to accurately measure many of the most abundant polar compounds.

### Organic sulfur compounds.

Six of the top 30 most abundant compounds in our environmental samples were organic sulfur compounds. These compounds fall into two general categories: sulfoniums ([SR_3_]^+^) and sulfonates ([RSO_3_]^–)^. We detected the well-studied sulfonium compound dimethylsulfoniopropionate (DMSP), though our methods likely underestimated the concentration due to compound instability in methanol-based extractions (49). Using our untargeted approach, we putatively identified two additional sulfonium compounds, dimethylsulfonioacetate (DMS-Ac) and 3-5-dimethylsulfonio-3-hydroxypentanoate (gonyol), as prominent peaks in our environmental samples. We later obtained standards that confirmed these identifications and enabled quantification that revealed gonyol as among our most abundant compounds with a particularly high concentration (up to 2.5 nM) in the NPSG depth profile (Fig. S7). Gonyol was named after the dinoflagellate Gonyaulax polyedra (50), and gonyol was present in high concentrations in all four dinoflagellate strains (81 to 196 mM) and in lower concentrations in the haptophytes (23 to 61 mM, Fig. S5 and S7; see also Table S6 at https://doi.org/10.5061/dryad.brv15dv8s). The environmental samples contained more DMS-Ac per unit carbon than culture samples, suggesting a source of this compound in the environment not reflected in the cultured phytoplankton (Fig. S3). Both of these compounds share structural similarity with DMSP and disrupt bacterial DMSP degradation pathways (51). Thus, predicting marine dimethyl sulfide (DMS) production from DMSP may be complicated by these highly abundant compounds. Although marine organic sulfur has gained much attention with regard to its massive inventory (52) and role in microbial processes (53), ours are the first observations of these understudied sulfoniums in natural marine systems.

10.1128/mSystems.01334-20.7 FIG S7 Gonyol and DMS-Ac spatial patterns in meridional transect (A), NPTZ depth profile (B), and NPSG depth profile (C). Intracellular concentrations of gonyol and DMS-Ac in applicable organisms (D). Note the different scales for the compound concentrations (C) and depths (between panels B and C). Download FIG S7, PDF file, 0.01 MB. Copyright © 2021 Heal et al. 2021 Heal et al. https://creativecommons.org/licenses/by/4.0/ This content is distributed under the terms of the Creative Commons Attribution 4.0 International license.

### Remaining unidentified compounds.

Many of the metabolites with interesting patterns across space and taxonomy could not be identified. For example, the mass feature I121.0684R10.7 has a *m/z* of 121.0684 and major peak in its MS^2^ fragmentation spectra of *m/z* 63.02703 (see Table S5 at https://doi.org/10.5061/dryad.brv15dv8s). This metabolite likely has the empirical formula of C_5_H_12_OS and was observed in 19 of the 21 phytoplankton species, attenuated with depth, and had a distinct maximum from 32 to 34°N in the meridional transect (see Table S5 at https://doi.org/10.5061/dryad.brv15dv8s). Unfortunately, none of the possible matches to these compounds have fragmentation data in the major mass spectral databases; without an identification, we cannot quantify this compound. It is very likely that within these unidentified compounds are more underappreciated compounds involved in the microbial loop—a fruitful endeavor for future oceanographers, mass spectrometrists, and biochemists alike.

### Conclusions.

The work here explores the small molecules within marine particulate organic matter which contribute to the dissolved organic matter pool after excretion, cell lysis, or sloppy feeding. Once in a dissolved form, other organisms in the environment may be able to use these compounds as the substrates as sources for carbon, nutrients, and energy (54, 55), if they have the required enzymatic machinery to access these resources. These small molecules may also act as chemical attractants or deterrents for organisms and therefore assist in shaping microbial communities. By directly observing small molecules in both field particulate material and cultured phytoplankton, we show that small molecules in natural marine systems are determined in part by the taxonomy of the phytoplankton community. This suggests that to access these pools of labile organic carbon, the wider microbial community must be adapted to phytoplankton community composition. By quantitatively contextualizing our metabolomics data sets, we uncover a rich set of compounds that likely fuel the microbial loop that have been previously overlooked. Cycling of organic matter thus depends both on the amount of primary productivity and on phytoplankton composition—“who” matters on a chemical level.

## MATERIALS AND METHODS

### Environmental sample collection.

Samples were collected for environmental metabolomics of particulate material at locations shown in Fig. 1. Samples for the NPSG depth profile were collected aboard the R.V. *Kilo Moana* cruise KM1513 on 31 July 2015 from four depths (15, 45, 75, and 125 m); we reported on these samples in a previous publication (16). Samples for the meridional transect were collected on cruise KOK1606 aboard the R.V. *Ka’imikai-O-Kanoloa* from 20 April to 2 May 2016, all at approximately 15 m. Samples for the NPTZ depth profile were collected during MGL1704 aboard the R.V. *Marcus Langseth* at seven depths between 30 and 250 m on 3 June 2017. At each sampling location and depth, single, duplicate, or triplicate filters were collected for environmental metabolomics, as previously described (16), using either Niskin bottles or the uncontaminated underway seawater intake. Table S3 in the supplemental material has summarized descriptions of the samples collected for metabolomics, with full description of each sample (including time of collection) in Table S4 at https://doi.org/10.5061/dryad.brv15dv8s. In short, samples (4 to 15 liters each) were collected into polycarbonate carboys, filtered onto 147-mm 0.2-μm polytetrafluoroethylene (PTFE) filters using a peristaltic pump, flash frozen in liquid N_2_, and stored at −80°C until extraction. In addition to our samples, we filtered duplicates of methodological blanks by filtering seawater through two 0.2-μm PTFE filters in series and used the second filter as the blank. This blank is especially important to parse metabolite signals from contaminants as well as compounds within the residual dissolved pool and salt matrix adsorbed during filtration.

### Pure cultures and sampling.

In addition to environmental samples, we analyzed metabolomes of cultured representatives of marine phytoplankton that were grown and analyzed on the same LC-MS system as previously presented (5). Media, light, and temperature were chosen for optimal growth of each species and are reported in reference 5. In short, axenic phytoplankton were cultured in controlled laboratory settings and harvested under exponential growth using a gentle vacuum filtration onto 47-mm Durapore filters (pore size, 0.2 *μ*m). Samples were flash frozen in liquid N_2_ and stored at −80°C until extraction. In addition to samples, medium blanks corresponding to each medium type were harvested and served as matrix blank to each corresponding phytoplankton sample. In order to estimate intracellular concentrations of metabolites, we used biovolume estimates from reference 5.

We also grew Nitrosopumilus maritimus strain SCM1 and harvested it under exponential growth. Pure culture of Marine Group I *Thaumarchaeota* Nitrosopumilus maritimus strain SCM1 was maintained in liquid mineral medium with 1 mM ammonia (56) at 30°C in the dark without shaking. The growth of *N. maritimus* was monitored by measuring nitrite production and cell abundance. Nitrite concentration was determined spectrophotometrically using the Griess reagent (57). Cell counts were determined using Moviol-SYBR green I staining protocol as previously reported (58) with a Zeiss epifluorescence microscope to count 15 random fields of view for each sample with 30 to 200 cells per field. Mid-exponential-phase cells were harvested using a gentle vacuum filtration on 0.22-*μ*m Durapore membrane filters (Millipore Co., MA, USA) and stored at −80°C until metabolite extractions. These archaea have a biovolume of approximately 0.023 *μ*m^3^ (59).

We estimated carbon contents for all the cultures from cellular volume (60), using an empirical relationship between flow cytometry-based cell size and PC (61), or using previous direct measurements (61–63). An abbreviated sample description is given in Table S3; full sample descriptions are in Table S7 (at https://doi.org/10.5061/dryad.brv15dv8s, including carbon estimates and the method used for each species).

### Additional oceanographic data.

Samples for particulate carbon were sampled and processed as in reference 64. Underway flow cytometry data were acquired and processed as in reference 61. Samples for pigment analysis were filtered onto GF/F filters (Whatman), stored in snap-cap tubes, wrapped in aluminum foil, and flash-frozen. Samples were analyzed for high-performance liquid chromatography (HPLC)-based measurements of total chlorophyll (monovinyl + divinyl), fucoxanthin, and other photosynthetic and photoprotective pigments. These analyses were made in the Oregon State University HPLC facility via a Waters 996 absorbance photodiode array detector in combination with a Waters 2475 fluorescence detector according to the protocol of reference 65.

### Homarine bioavailability experiment.

To test if homarine was as bioavailable as trigonelline in marine systems, we cultured the model marine heterotrophic bacterium Ruegeria pomeroyi DSS-3 under different primary carbon sources and observed its cell density. DSS-3 was streaked to isolation on 1/2 YTSS agar plates (1.25 g tryptone, 2 g yeast extract, 10 g sea salts, 8 g agar per 500 ml MQ water) from frozen glycerol stocks at room temperature for 3 days. A single colony was inoculated into artificial L1-bac seawater medium (described below) supplemented with acetate (final concentration of 50 nM). This culture was grown overnight at room temperature at 200 rpm at 30°C. Next, a 96-well plate was prepared with 90 μl of fresh medium described above (without additional carbon) in all wells. In 8 wells, we added 5 μl of the overnight inoculum and 10 μl of water (no additional carbon treatment). In 8 wells, we added 15 μl of water and no inoculum (negative control). In the remaining wells, we added 5 μl of the overnight inoculum and 10 μl of either acetate, homarine, or trigonelline (all at 100 nM carbon, *n *= 8 for each, acetate serving as positive control). Plates were covered in a breathable sealing membrane (Breathe-Easy) and placed into a plate reader (BioTek Synergy H1MF). Cultures were grown at 30°C, shaken every 2 min for 3 s, and monitored via absorbance at 600 nm every 2 min (immediately after shaking).

Artificial L1-bac seawater medium was prepared using MQ water with 28 g Sigma sea salts, trace and macronutrients based on the recipe from the National Center for Marine Algae and Microbiota (without silica), nitrogen, and vitamins as in reference 18; Sigma M5550 MEM essential amino acids (1:1,000 dilution); and Sigma M7145 MEM nonessential amino acids (1:2,000 dilution). Salt water was autoclaved in combusted borosilicate glass containers, and all additions were made from filter-sterilized stocks. Final medium was filter sterilized using a 0.22-*μ*m polyvinylidene difluoride (PVDF) membrane bottle top filter.

### Metabolite data acquisition.

Metabolites were extracted as previously described (16). Briefly, filters were bead-beaten three times in 30-s bursts over 30 min (kept at −20°C between bursts) in 1:1:2 methanol-water-dichloromethane and separated into two fractions: a polar aqueous extract (methanol and water extractable) and an organic extract (dichloromethane extractable). We used the same internal standard suite at the same injection concentrations as in the work of Boysen et al. (16) to train normalization and monitor instrument stability. After drying under clean N_2_, all samples were reconstituted in 400 μl water.

The polar fraction of this extract was analyzed on both reversed-phase (RP) and hydrophilic interaction chromatography (HILIC) using the same solvents, columns, and gradients as previously reported (16). We diluted the KOK1606 samples (1 part sample to 2 parts water) and MGL1704 samples (1 part sample to 1 part water), which helped with signal stability over the course of the runs. Internal standards were added during the dilution step and were the same concentrations in all analyzed samples to aid in quantitative comparisons between sample sets. We injected 2 μl of sample onto the column for HILIC analysis and 5 μl (for environmental samples) or 15 μl (for culture samples) for RP analysis.

Both LC configurations (RP and HILIC) were analyzed on a Thermo Q-Exactive (QE) mass spectrometer in full scan mode for quantitative data or data-dependent acquisition (DDA) for fragmentation. Full scan analyses were conducted as in the work of Boysen et al. (16); pooled samples were run in DDA mode for MS^2^ fragmentation as described in the work of Heal et al. (20).

### Metabolomic data processing.

To compare our field data with our culture data, we used our untargeted data from the meridional transect (36 surface samples) as our template to examine the other sample sets. To do this, we used an established untargeted metabolomics approach (detailed below) to acquire a list of curated, dereplicated, and high-quality mass features. With this curated list, we then searched for the same mass features in the remaining field and culture sample sets. This allowed us to compare relative abundances of these mass features within each sample set, with high confidence in the shared identity of these compounds between sample sets.

Untargeted metabolomics data from transect samples were converted with MS Convert (66) and processed through XMCS (67–69), using the same parameters for XCMS and methodological blank filtering as previously reported (20). Next, we normalized for obscuring variation (nonbiological variability inherent to LC-MS analysis) using B-MIS normalization (16). As in the work of Heal et al. (20), we disregarded peaks that did not demonstrate acceptable replicability in the pooled samples (coefficient of variance > 30%); we also removed peaks that showed greater average variability between biological replicates than over the whole sample set as in previous work (4).

In untargeted metabolomics, multiple mass features can correspond to one metabolite due to natural abundance isotopes, adducts, or multiply charged ions. As in the work of Heal et al. (20), to avoid putting extra statistical weight on these isotopes and adducts, we identified mass features that were likely ^13^C, ^15^N, or ^34^S isotopologues of other mass features. We extended this search to include adducts of Na^+^, NH_4_^+^, K^+^ (for positive ionization), and Cl^–^ (for negative ionization), as well as for doubly charged ions of mass features whose M+H ion was present. We performed these searches within each 3-s (for RP) or 6-s (for HILIC) corrected retention time window and discarded these mass features from downstream statistical analyses.

For the largest 200 peaks in our HILIC analysis (positive and negative analyzed separately) and RP analysis, we exported the *m/z* and retention time information to Skyline (70) for closer inspection. XCMS peak picking algorithms assume a normal Gaussian shape for peaks (67–69), which often results in poor integrations for compounds that do not achieve this shape during chromatographic separation; these peaks are often removed during our coefficient of variance (CV) filter or manual peak quality verification. Therefore, we also imported a list of compounds we regularly target (see reference 16 for full list of standards) and manually integrated these compounds in each of the samples (first removing compounds that were picked during the peak picking step). In Skyline (70), we integrated these peaks (both the untargeted and known compounds) in the transect data set since XCMS often results in imperfect integrations that can introduce nonbiological variability to metabolite abundances (71). Next, we eliminated mass features that were not present in at least 50% of the transect samples and also removed peaks that were not (on average) three times larger than the matrix blank. These stringent filters in the transect data set allowed us to use a culled number of high-quality mass features that are common in surface seawater particles as a fingerprint of metabolite pools. In all, we obtained 149, 74, and 90 high-quality, manually integrated peaks in HILIC-positive ionization (HILICPos), HILIC-negative ionization (HILICNeg), and RP, respectively. For these quality mass features, we searched for corresponding MS^2^ scans in the data-dependent acquisition (DDA) files and applied a filter to remove low-abundance fragments in the exact manner as reported in reference 20.

With this list of high-quality mass features (referred to in the text as metabolites), we extracted the exact masses and integrated peaks at the same retention times in the two other environmental data sets (NPSG and NPTZ depth profiles) and the culture data sets. We also integrated our internal standards (in exact concentrations as in reference 16) and performed B-MIS normalization (16) across our environmental data sets, which minimizes the variability present during analysis (not biological variability). This resulted in adjusted areas of each compound in each sample that are quantitatively comparable within each sample set (but not between). Since the phytoplankton data sets are not in a consistent matrix and were analyzed in several different batches, we did not attempt to use B-MIS to normalize across the organisms. Instead, we kept the raw peak area, normalized it to the biovolume analyzed, and made semiquantitative comparisons on the log_10_-transformed biovolume-normalized peak areas. The log_10_ transformations ensure that only large differences are evaluated as contributing to variability between samples, well beyond matrix variability or instrument performance.

As in reference 20, we used the ranking system outlined in reference 72, to attempt to identify the quality mass features present in these sample sets in an automated fashion. We searched an internal database of compounds with known exact *m/z* and retention time on the LC-MS configurations used in the lab (found at https://github.com/IngallsLabUW/Ingalls_Standards), publicly available MS/MS^2^ spectral databases (73–77), and compounds in the KEGG database (78, 79) (based only on *m/z*).

### Calculating concentrations.

Commercially available standards were analyzed in the same batch as each of the three environmental data sets. For this subset of compounds, we calculated absolute concentrations, similar to previous work (12, 21, 41). In short, we applied the following calculation for each analyte:

$$\text{Concentration}\;\text{=}\;\frac{\text{Area}}{\text{RF}}\text{×}\frac{{\text{Vol}}_{\text{reconst}}}{{\text{Vol}}_{\text{filtered}}}\text{×}\frac{\text{1}}{{\text{(RF}}_{\text{ratio}}\text{)}}$$

where RF is the response factor $\frac{(\text{Area}}{\text{concentration})}$ of each compound at known concentration in water. Standards are run before and after each run on each instrument; therefore, an RF for each compound is obtained within each batch. Vol_reconst_ is the volume into which the samples were reconstituted; Vol_filtered_ is the volume filtered in the field (for environmental samples) or the total estimated biovolume collected (for culture samples); RF_ratio_ is the $\frac{{\text{RF}}_{\text{matrix}}}{{\text{RF}}_{\text{water}}}$ of these compounds in a matrix of marine particulates (as described in the work of Boysen et al. [16]); we calculated RF_ratio_ using samples from the transect data set which were applied throughout. We calculated an RF_ratio_ as in reference 16 using a representative environmental matrix sample. Values for Vol_filtered_ for each sample are reported in Tables S4 and S7 (both at https://doi.org/10.5061/dryad.brv15dv8s); Vol_reconst_ was 400 μl for each sample.

Several compounds were identified in the transect data set and purchased and analyzed using the same LC-MS method at a later date, which we quantified using the same approach as in reference 41. Because the RF for each compound can vary substantially between analytical runs, we used a relative response factor (RF_relative_) to estimate RF and calculate the concentrations of these compounds in earlier runs. To calculate RF_relative_, we matched compounds with a standard that had been analyzed in all sample sets that share the same column, ionization state, and some structural similarity (matched standard). For instance, for the compound DMS-Ac, we matched it to another dimethylated sulfonium zwitterion, DMSP. After the samples were analyzed, we analyzed these new standards and the other standards on the same LC-MS setup as our sample set and calculated RF_relative_ using the following formula:

$${\text{RF}}_{\text{relative}}\;=\;\frac{{\text{RF}}_{\text{analyte}}}{{\text{RF}}_{\text{matched standard}}}$$

Then, we used this RF_relative_ and the RF_matched standard_ to calculate the concentration of the analyte from earlier runs. For a full explanation for how each compound was quantified in each sample set and for the matched standard used for each compound (when necessary), see Table S11 at https://doi.org/10.5061/dryad.brv15dv8s.

### Statistical approaches.

For multivariate statistics on environmental samples, peak areas (adjusted via B-MIS for instrumental variability and normalized to water volume filtered) were standardized to the total peak area observed for each mass feature across each sample set. For each mass feature in the cultured organisms, log_10_-transformed peak areas were standardized to the maximum log_10_ peak areas observed across all cultured organisms. We used two different multidimensional approaches on these data sets, both nonmetric to accommodate for the high variable-to-sample ratio and nonnormal distribution of peak areas in our data sets. This prevents overfitting, which can be a problem in other multidimensional approaches in metabolomics (80). We used a nonmetric dimensional scaling (NMDS) analysis (81) based on a Euclidean distance matrix of standardized peak areas to visualize overall metabolic differences between samples along our transect samples. We assessed dimensionality of the NMDS by examining a scree plot and calculated the probability with a Monte Carlo permutation which resulted in a low-stress ordination. We accompanied this with an analysis of similarities (ANOSIMS) (82) to discern differences between the oceanographic regimes we sampled as well as time of sampling. Data transformation, standardization, NMDS, and ANOSIMS statistics were performed in R using the vegdist (v2.4-2) or vegan (v2.4-2) packages.

Next, we employed a *k*-medoids-based clustering approach (83), which aggregates metabolites based on patterns across samples. We performed this clustering on the combined culture data sets and on the three environmental sample sets separately (four total *k*-medoids analyses) using the clara function in the cluster package (v2.1.0) in R. This nonsupervised clustering technique is exclusive and nonhierarchical and assigns each mass feature into one cluster, or mode. The metabolites within each mode have similar patterns of abundance across samples. We chose the appropriate number of modes for each sample set by selecting the mode number that resulted in a local maximum average silhouette width between samples.

Finally, we investigated whether the resulting modes of metabolites from the four separate *k*-medoids analyses shared metabolites beyond a random assignment. Essentially, we asked if the patterns in the 2016 transect samples could be explained in part by patterns in metabolites across the available culture data or could be recapitulated in the depth profile sample sets. To test for overrepresented sharing of metabolites between modes, we used a Monte Carlo resampling technique to simulate the random frequency of shared metabolites using 1,000 permutations. We then compared the observed frequency of shared metabolites to the permutations to estimate the *P* value of our observed shared metabolites. To assign a compound to a metacluster (as noted in Table S6 at https://doi.org/10.5061/dryad.brv15dv8s), an individual compound must be assigned to the organism mode rooting each cluster and to at least 2 of the modes from the environmental samples within the metacluster.

### Data availability.

For all data analysis, we used R v4.0.0. Codes for figures, tables, and data analysis are found at https://github.com/kheal/Gradients1_SemiTargeted3. Raw data for metabolomics samples are deposited at Metabolomics Workbench (https://www.metabolomicsworkbench.org/): cultures are project ID ST001514, meridional transect samples are project ID ST001410, NPSG depth profile is project ID ST001372, and NPTZ depth profile is project ID ST001409. Project IDs for environmental samples also listed in Table S4 at https://doi.org/10.5061/dryad.brv15dv8s.
