# Correction for Heal et al., “Marine Community Metabolomes Carry Fingerprints of Phytoplankton Community Composition”

**DOI:** 10.1128/msystems.01086-22

**Published:** 2023-03-20

**Authors:** Katherine R. Heal, Bryndan P. Durham, Angela K. Boysen, Laura T. Carlson, Wei Qin, François Ribalet, Angelicque E. White, Randelle M. Bundy, E. Virginia Armbrust, Anitra E. Ingalls

## AUTHOR CORRECTION

Volume 6, no. 3, e01334-20, 2021, https://doi.org/10.1128/mSystems.01334-20. After publication, we discovered that the authentic standard of homarine that we purchased from a commercial supplier (Santa Cruz Biotechnology, lot B1916) was contaminated with picolinic acid. This contamination resulted in an overstatement of the actual homarine concentration in these samples. The correct values for the homarine concentration are 1/25 of the previously published values. This correction is based on a comparison of our original standard with both a commercially purchased isotope labeled standard and a newly purchased homarine standard. The recalculated values for homarine are reflected in the corrections below.

Page 1, abstract, line 7 from bottom: “3%” should read “0.25%.”

Page 8: Lines 2–7 should read as follows. “…The combined concentration of the identified metabolites (85 of the 313 total) ranged from 41 to 178 nM particulate carbon in the surface transect samples (Fig. S1B; Fig. S4; see Table S9 at https://doi.org/10.5061/dryad.brv15dv8s). This corresponds to 1.8% (±0.6%) to 2.5% (±0.4%) of the particulate carbon pool and 2.0% (±0.7%) to 3.3% (±1%) of the particulate nitrogen pool across this transect (see Table S9 at https://doi.org/10.5061/dryad.brv15dv8s)….”

10.1128/msystems.01086-22.1 FIG S1 Latitudinal trends in particulate carbon and fucoxanthin. Download FIG S1, PDF file, 0.03 MB. Copyright © 2023 Heal et al. 2023 Heal et al. https://creativecommons.org/licenses/by/4.0/ This content is distributed under the terms of the Creative Commons Attribution 4.0 International license.

10.1128/msystems.01086-22.2 FIG S3 All identified and quantified compounds. Download FIG S3, PDF file, 0.01 MB. Copyright © 2023 Heal et al. 2023 Heal et al. https://creativecommons.org/licenses/by/4.0/ This content is distributed under the terms of the Creative Commons Attribution 4.0 International license.

10.1128/msystems.01086-22.3 FIG S4 The 18 most abundant metabolites in environmental samples. Download FIG S4, PDF file, 0.03 MB. Copyright © 2023 Heal et al. 2023 Heal et al. https://creativecommons.org/licenses/by/4.0/ This content is distributed under the terms of the Creative Commons Attribution 4.0 International license.

Page 8: Lines 11 and 12 should read as follows. “…In the NPTZ depth profile, we quantified 12.6 to 514 nM particulate carbon in the metabolite pool, corresponding to a rough estimate of 5% of the particulate….”

Page 8, line 14: “approximately 3.7%” should read “approximately 3%.”

Page 8: Lines 8–12 from bottom should read as follows. “…The metabolite homarine (*N*-methylpicolinic acid) was present at 0.02 to 2.7 nM in marine particles and represented up to 0.25% of the total PC pool in our transect samples (Fig. 4 and 6; Fig. S5; see Table S10 at https://doi.org/10.5061/dryad.brv15dv8s)….”

10.1128/msystems.01086-22.4 FIG S5 All identified compounds. Download FIG S5, PDF file, 0.01 MB. Copyright © 2023 Heal et al. 2023 Heal et al. https://creativecommons.org/licenses/by/4.0/ This content is distributed under the terms of the Creative Commons Attribution 4.0 International license.

Page 8, lines 4–6 from bottom: The following passage should be deleted. “For example, other studies have shown that homarine in marine particles is less abundant than the compatible solute glycine betaine (GBT) (12, 39), contrasting with our findings.”

Page 9: Fig. 4 should appear as shown on next page.

**Figure:**
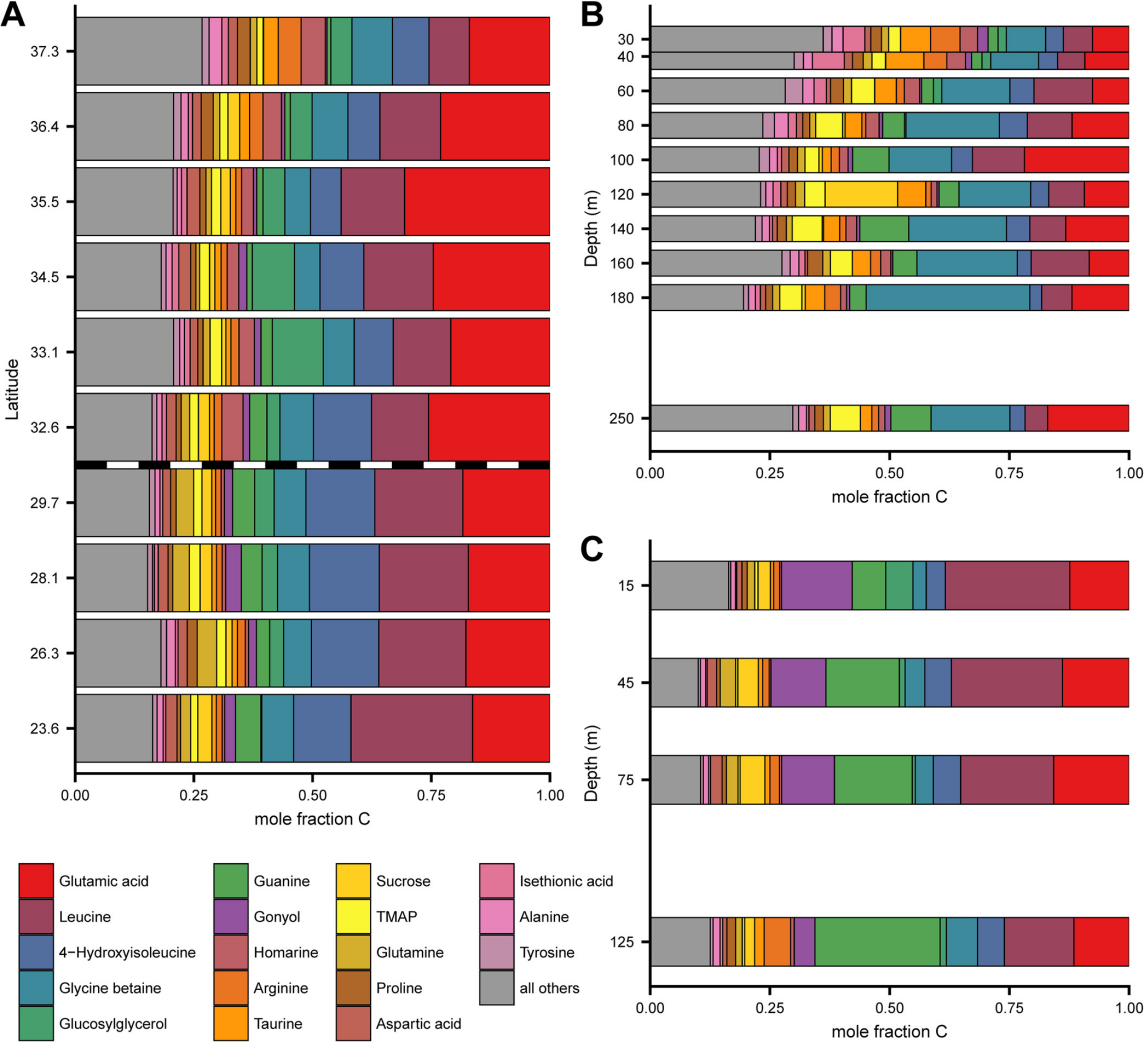


Page 9: Lines 5–7 should read as follows. “In our cultured isolates, we detected homarine in both *Synechococcus* strains (intracellular concentration up to 18 mM), four of six surveyed diatoms (0.02 to 2.2 mM), and one strain of *Emiliania huxleyi* (a haptophyte, at 0.15 mM) (Fig. 6D; see also Table S8 at https://doi.org/10.5061/dryad.brv15dv8s)….”

Page 9: Lines 2–5 from bottom should read as follows. “We estimated that homarine was 0.2% of the particulate carbon within *Synechococcus* strain WH8102. *Synechococcus* has been estimated to contribute 10% to 20% of global ocean net primary production at approximately 8 Gt C per year (46); by extrapolation this suggests up to 0.02 to 0.04%….”

Page 10: Fig. 5 should appear as shown on next page.

**Figure:**
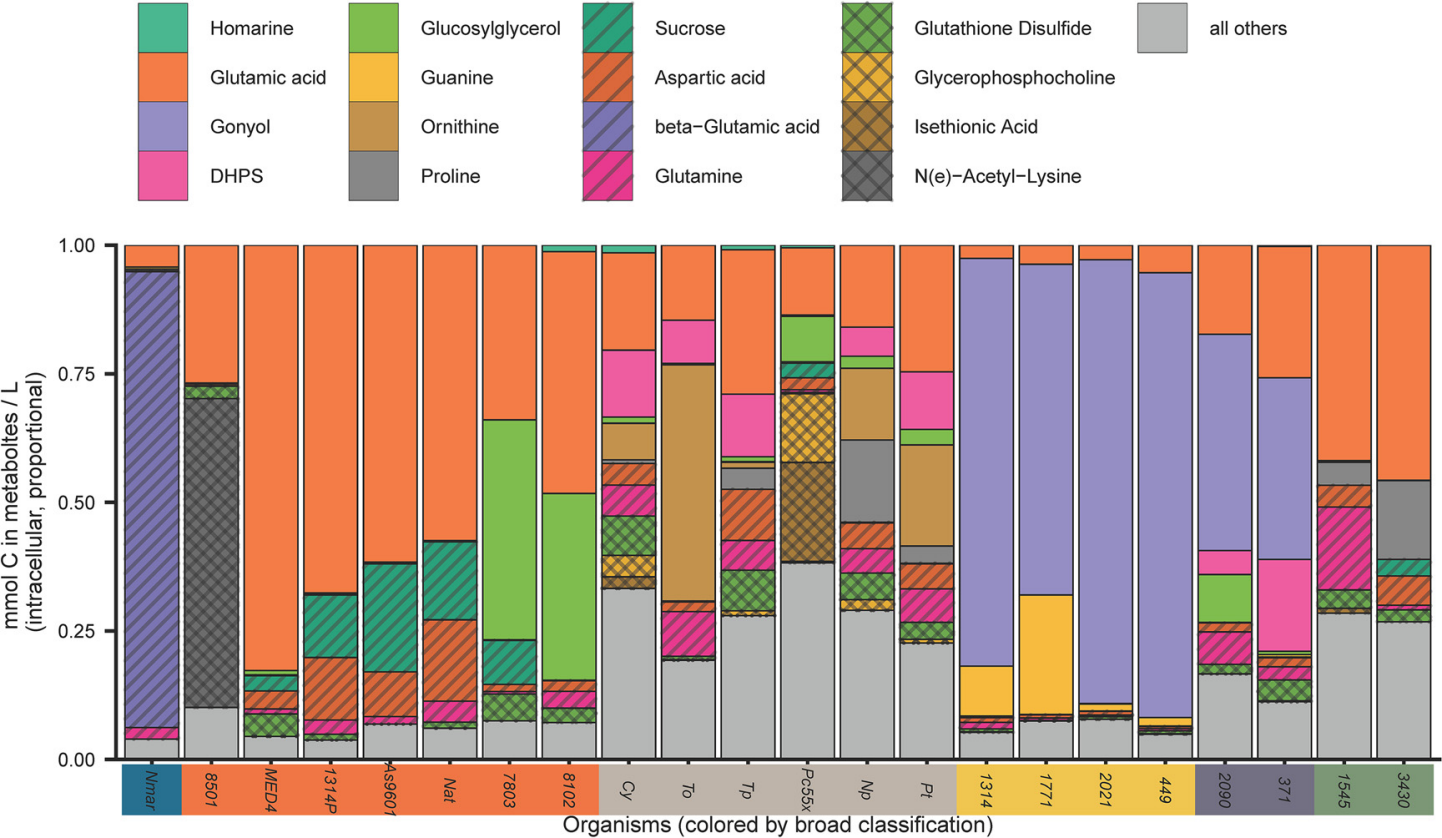


Page 10, line 4: “(4 to 5 mM)” should read “(about 0.2 mM).”

Page 10, line 7: “about 2%” should read “about 0.2%.”

Page 10, line 14: “(average 14.3 nM) than the NPSG (average 1.85 nM)” should read “(average 0.6 nM) than the NPSG (average 0.07 nM).”

Page 11: Fig. 6 should appear as shown below.

**Figure:**
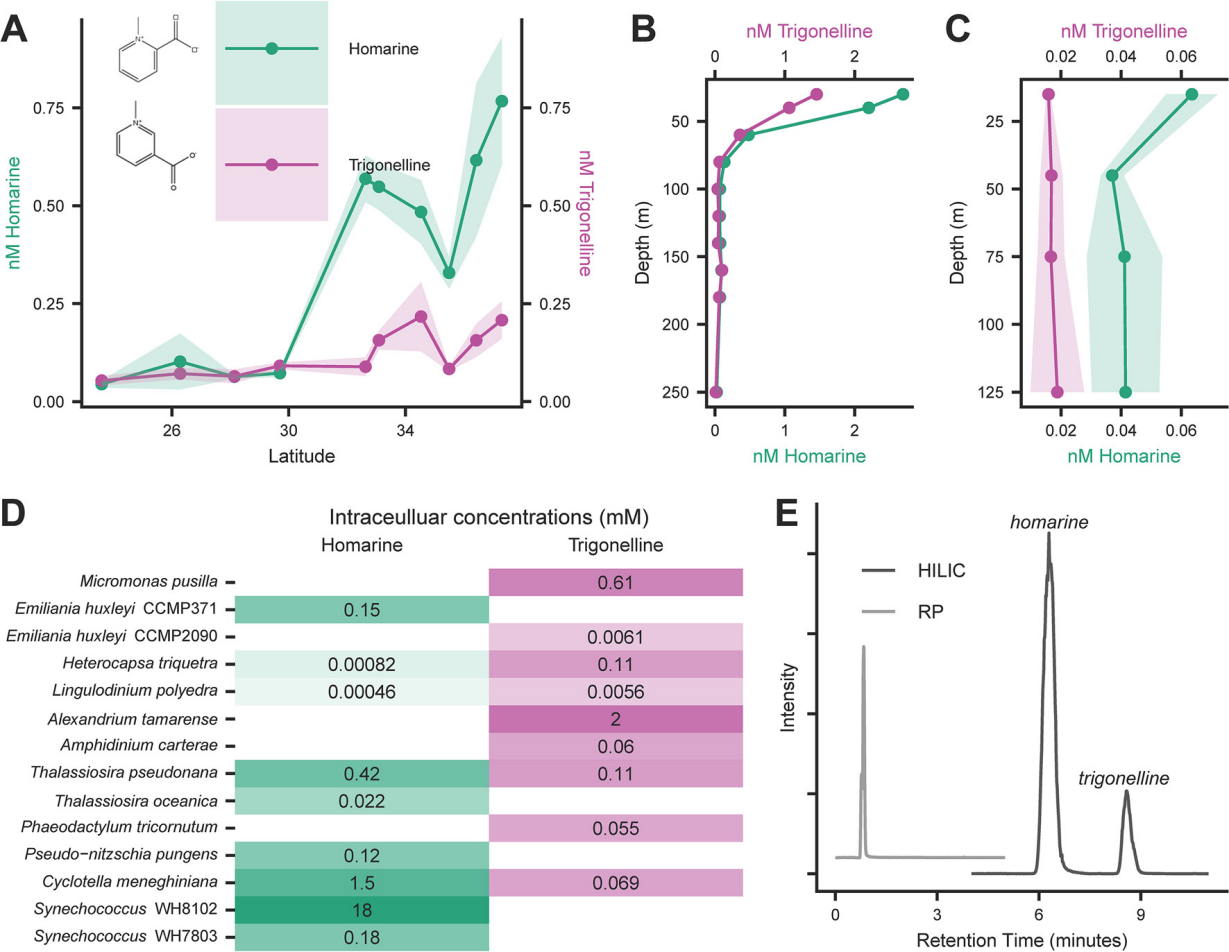


Page 11, lines 6–8: The following passage should be deleted. “Supporting the differential catabolism of homarine and trigonelline, we saw that the model marine heterotrophic bacterium Ruegeria pomeroyi DSS-3 was not able to grow on homarine as effectively as trigonelline (Fig. S6).”

Pages 13 and 14: The subsection “Homarine bioavailability experiment” should be deleted.

Supplemental material: Figs. S1, S3, S4, and S5 should appear as in the versions posted with this correction. Figure 6 should be deleted.

