# Supplementary figures and images for: Marine Community Metabolomes Carry Fingerprints of Phytoplankton Community Composition
Source: mSystems. 2021 May 4;6(3):e01334-20. doi: 10.1128/mSystems.01334-20 (PMC8269262; doi:10.1128/mSystems.01334-20)

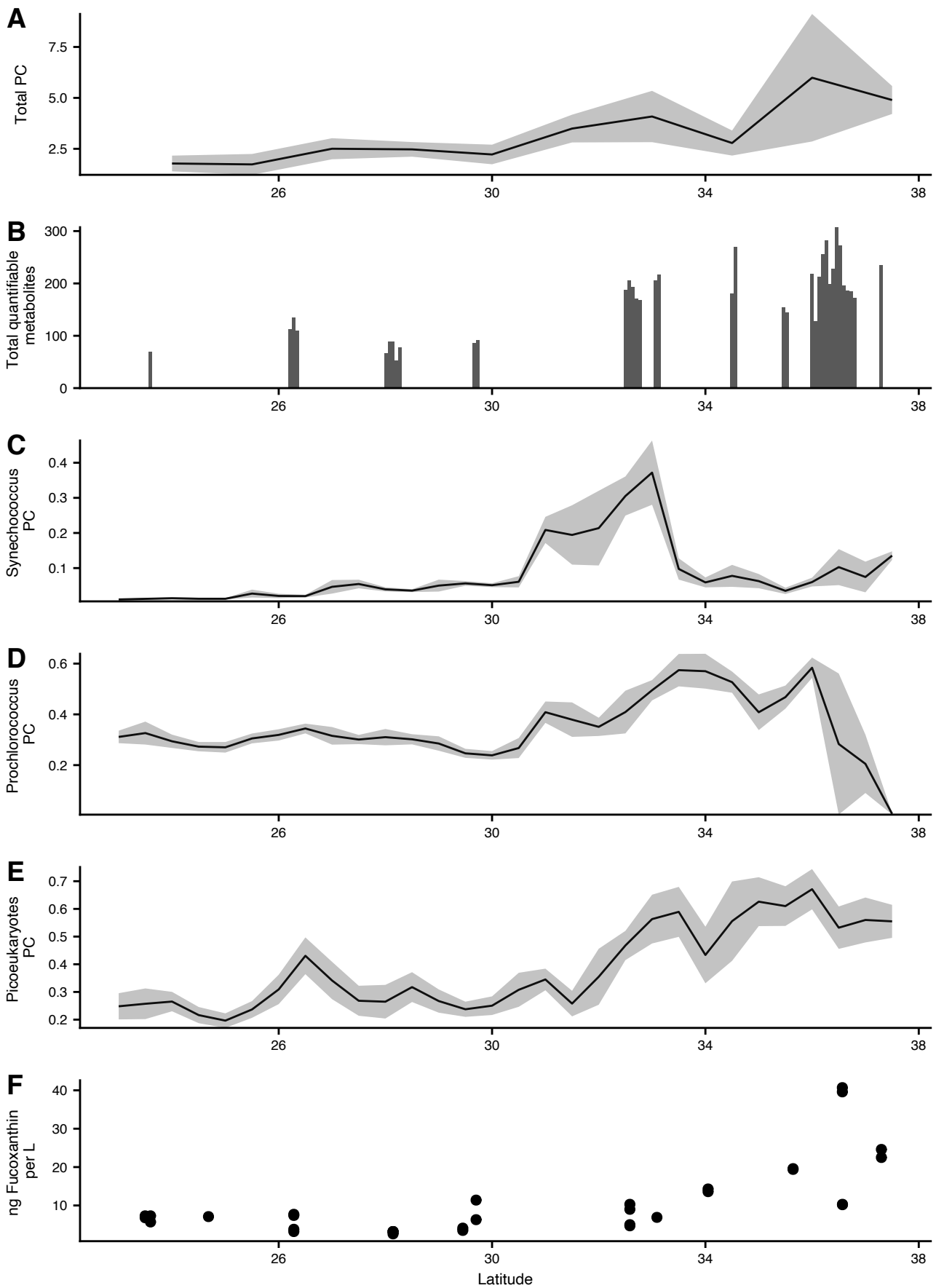

Supplement: FIG S1 [file msystems.01334-20-sf001.pdf]

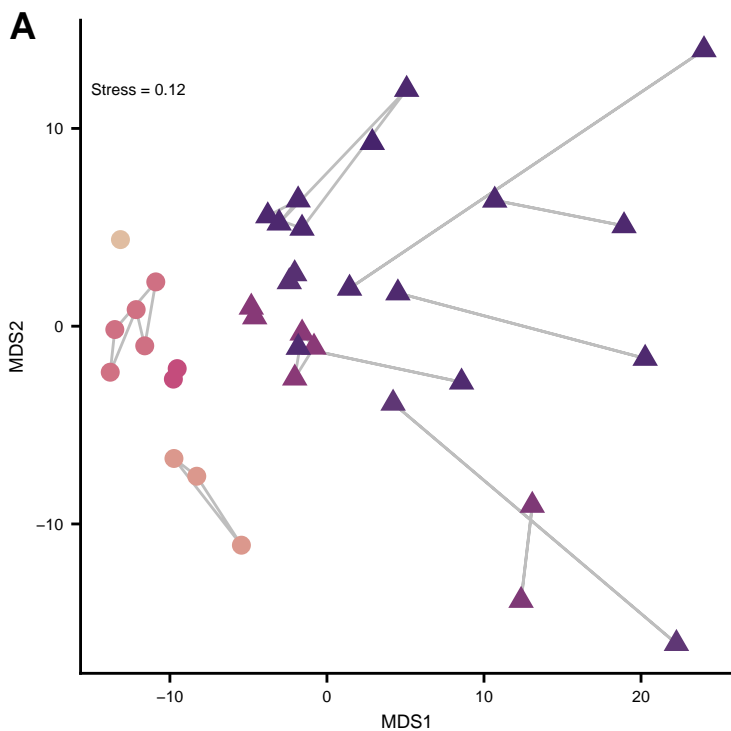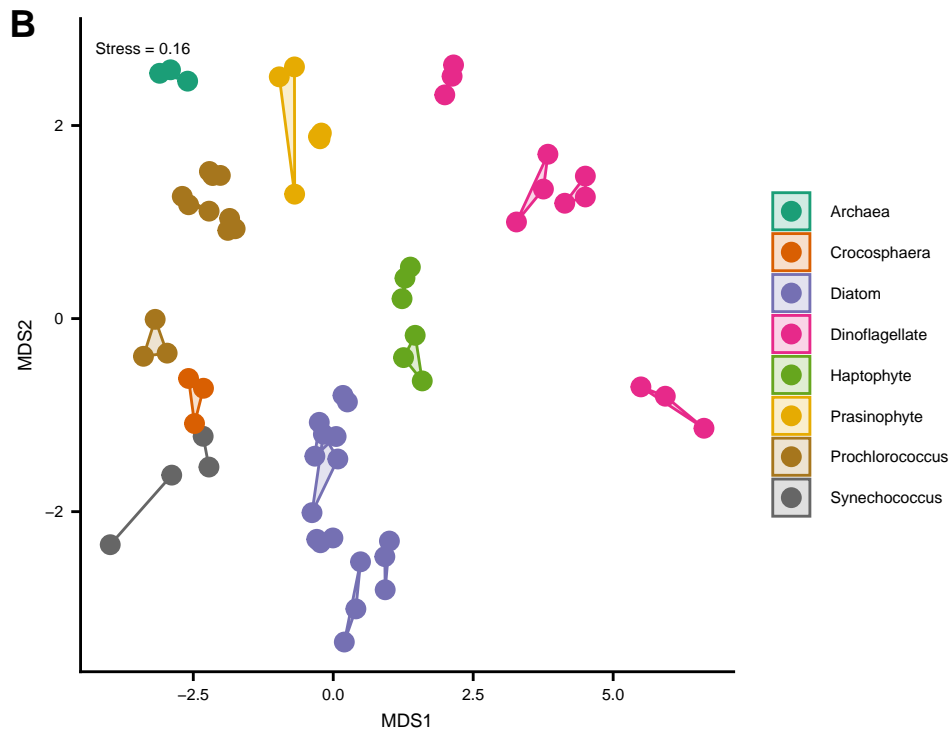

Supplement: FIG S2 [file msystems.01334-20-sf002.pdf]

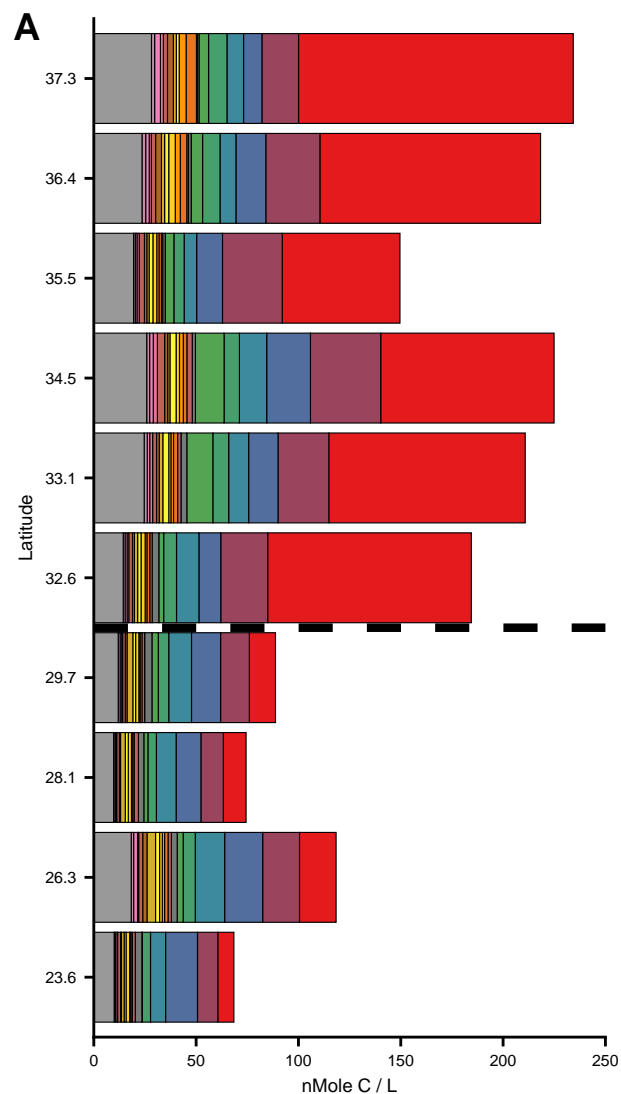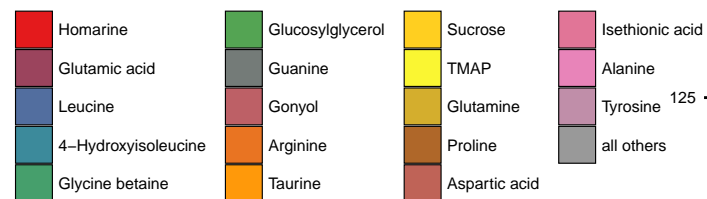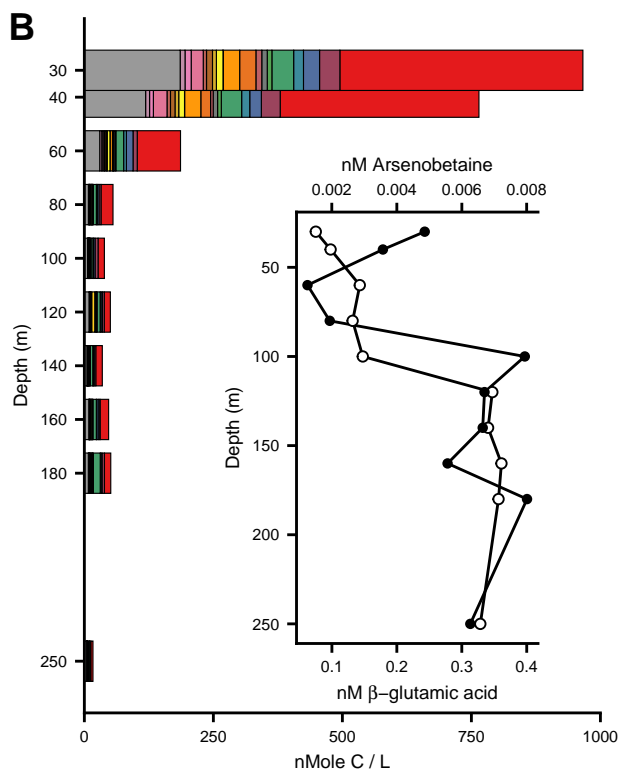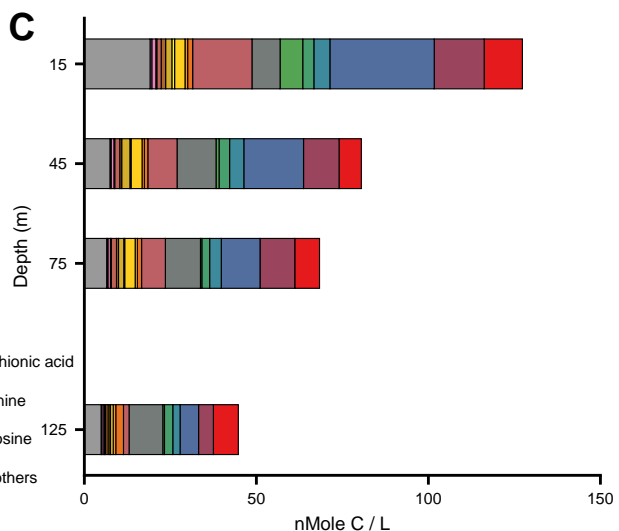

Supplement: FIG S4 [file msystems.01334-20-sf004.pdf]

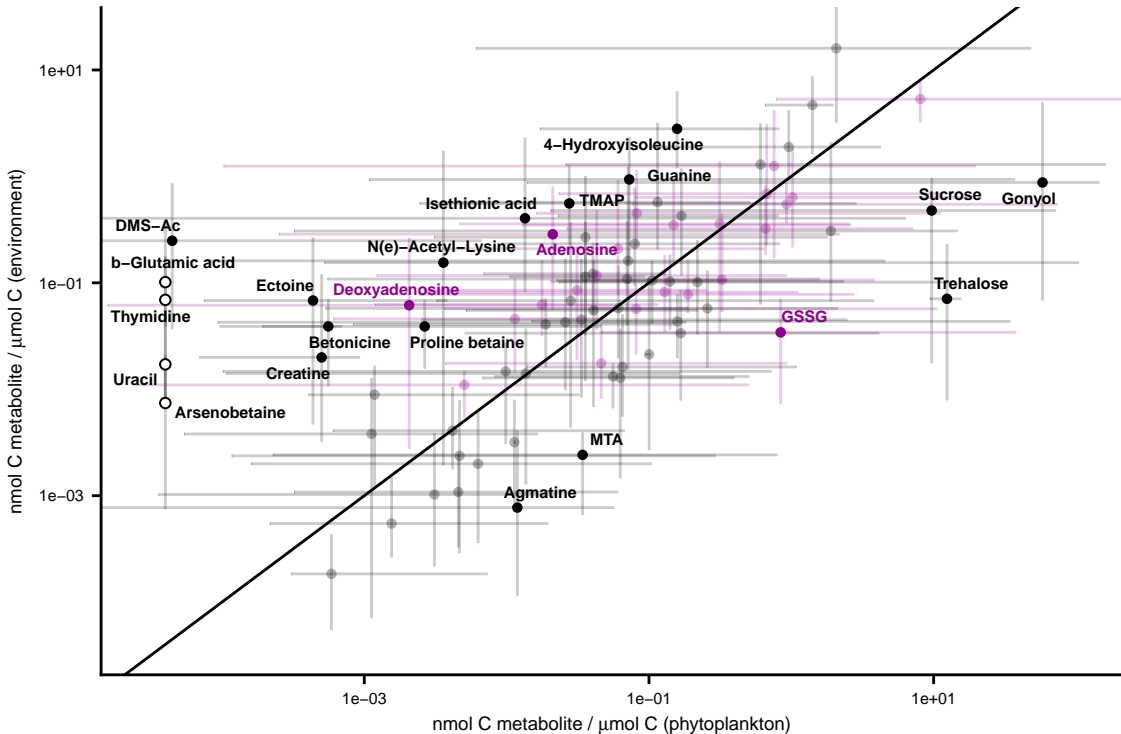

Supplement: FIG S3 [file msystems.01334-20-sf003.pdf]

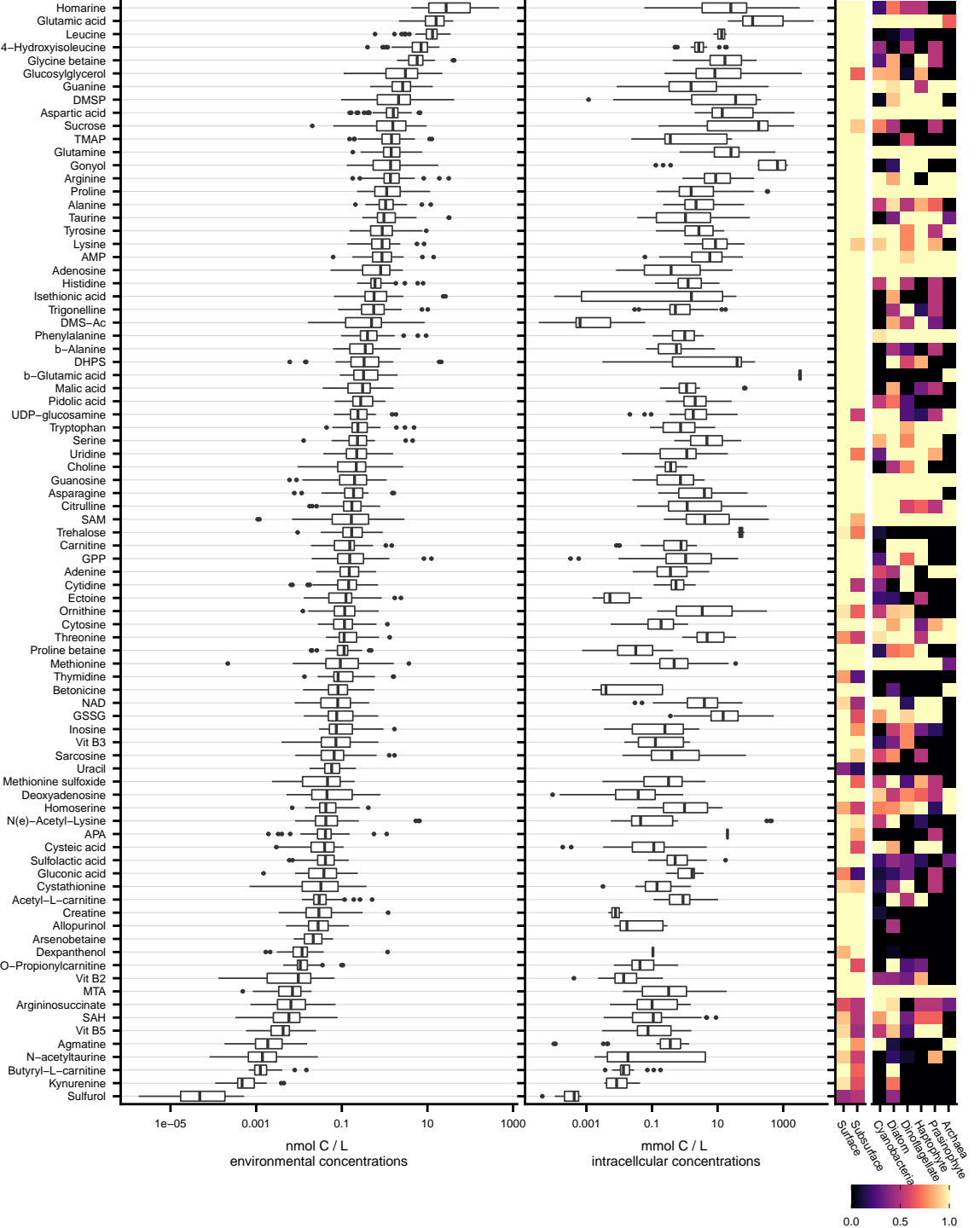

Supplement: FIG S5 [file msystems.01334-20-sf005.pdf]

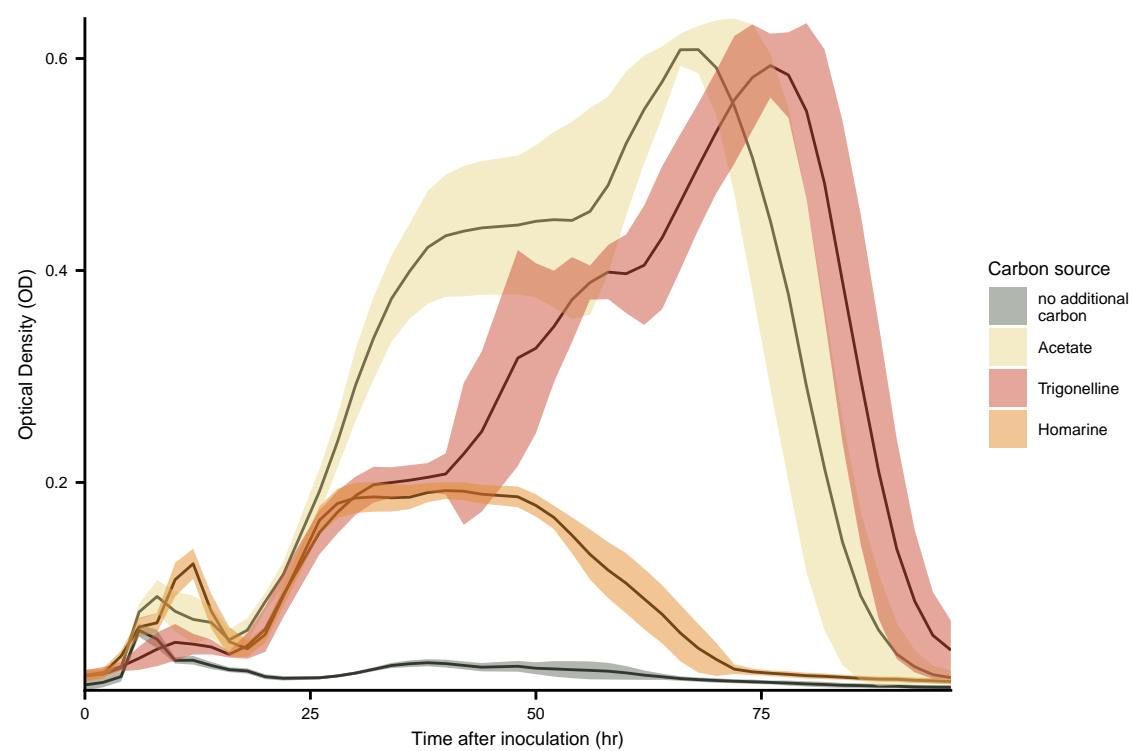

Supplement: FIG S6 [file msystems.01334-20-sf006.pdf]

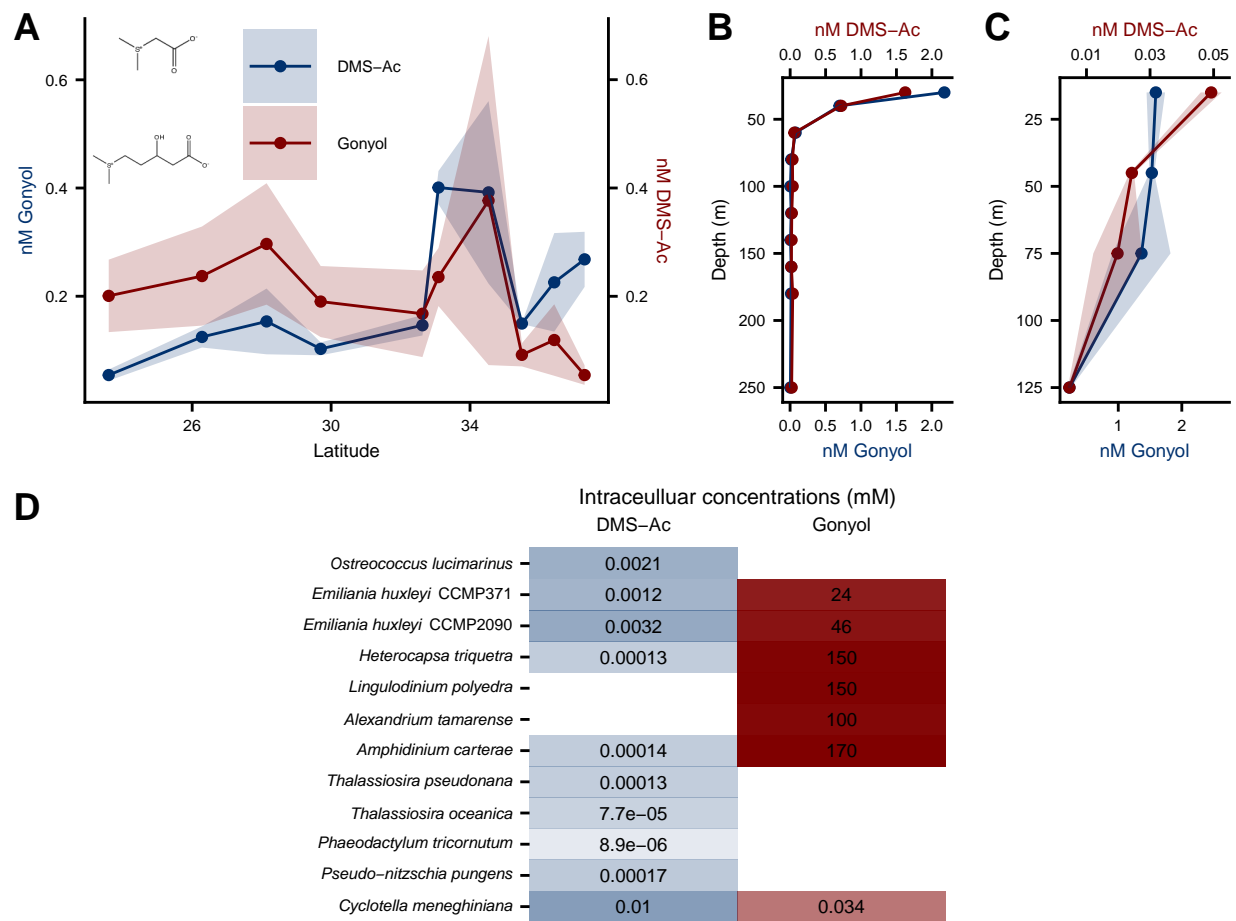

Supplement: FIG S7 [file msystems.01334-20-sf007.pdf]
