# Supplementary material for: Marine Community Metabolomes Carry Fingerprints of Phytoplankton Community Composition
Source: mSystems. 2021 May 4;6(3):e01334-20. doi: 10.1128/mSystems.01334-20 (PMC8269262; doi:10.1128/mSystems.01334-20)
Supplement: TABLE S1 [file msystems.01334-20-st001.pdf]

Cruise ID	Depth (m)	*n*	Date	Latitude	Longitude	Vol (L)	Collection method	Environmental regime
KM1513	15.00	3	2015-07-31	24.55	-156.33	11.00	niskin	NPSG
KM1513	45.00	3	2015-07-31	24.55	-156.33	9.00	niskin	NPSG
KM1513	75.00	3	2015-07-31	24.55	-156.33	13.00	niskin	NPSG
KM1513	125.00	3	2015-07-31	24.55	-156.33	12.00	niskin	NPSG
KOK1606	15.00	2	2016-04-21	23.60	-157.96	10.00	niskin	NPSG
KOK1606	15.00	3	2016-05-01	26.28	-158.00	10.00	niskin	NPSG
KOK1606	15.00	5	2016-04-22	28.14	-158.00	11.00	niskin	NPSG
KOK1606	15.00	2	2016-04-23	29.45	-158.04	15.00	underway	NPSG
KOK1606	15.00	2	2016-05-01	29.70	-158.00	10.00	niskin	NPSG
KOK1606	15.00	2	2016-04-24	30.40	-157.99	15.00	underway	NPSG
KOK1606	15.00	5	2016-04-24	32.63	-158.00	11.00	niskin	NPTZ
KOK1606	15.00	2	2016-04-30	33.09	-158.00	10.00	niskin	NPTZ
KOK1606	15.00	2	2016-04-25	34.53	-158.00	15.00	underway	NPTZ
KOK1606	15.00	2	2016-04-26	35.49	-158.01	13.00	underway	NPTZ
KOK1606	15.00	2	2016-04-29	36.22	-158.00	10.00	underway	NPTZ
KOK1606	15.00	2	2016-04-29	36.30	-157.99	10.00	underway	NPTZ
KOK1606	15.00	2	2016-04-29	36.37	-157.97	10.00	underway	NPTZ
KOK1606	15.00	2	2016-04-29	36.46	-157.96	10.00	underway	NPTZ
KOK1606	15.00	2	2016-04-29	36.57	-158.00	10.00	niskin	NPTZ
KOK1606	15.00	4	2016-04-27	36.57	-158.00	11.00	niskin	NPTZ
KOK1606	15.00	2	2016-04-26	37.30	-158.00	10.00	niskin	NPTZ
MGL1704	30.00	1	2017-06-03	41.42	-158.00	4.00	niskin	NPTZ
MGL1704	40.00	1	2017-06-03	41.42	-158.00	4.00	niskin	NPTZ
MGL1704	60.00	1	2017-06-03	41.42	-158.00	4.00	niskin	NPTZ
MGL1704	80.00	1	2017-06-03	41.42	-158.00	4.00	niskin	NPTZ
MGL1704	100.00	1	2017-06-03	41.42	-158.00	4.00	niskin	NPTZ
MGL1704	120.00	1	2017-06-03	41.42	-158.00	4.00	niskin	NPTZ
MGL1704	140.00	1	2017-06-03	41.42	-158.00	4.00	niskin	NPTZ
MGL1704	160.00	1	2017-06-03	41.42	-158.00	4.00	niskin	NPTZ
MGL1704	180.00	1	2017-06-03	41.42	-158.00	4.00	niskin	NPTZ
MGL1704	250.00	1	2017-06-03	41.42	-158.00	4.00	niskin	NPTZ

TABLE S1 Summary of samples collected and analyzed in this study.
