# Supplementary material for: Marine Community Metabolomes Carry Fingerprints of Phytoplankton Community Composition
Source: mSystems. 2021 May 4;6(3):e01334-20. doi: 10.1128/mSystems.01334-20 (PMC8269262; doi:10.1128/mSystems.01334-20)
Supplement: TABLE S2 [file msystems.01334-20-st002.pdf]

Region	Latitude (°N)	SST (°C)	SSS	N+N (μM)	PO_4 (μM)	Chl (mg m^{-3})
NPSG	23.54–29.7	19.8–24	35.1–35.3	dl–0.002	0.02–0.05	0.01–0.11
NPTZ	32.63–37.3	11.4–17.1	34.1–34.7	0.06–5.87	0.07–0.51	0.16–0.37

TABLE S2 Summary of physical and chemical parameters on April 2016 cruise. Reprinted with permission from (28).
