# Supplementary material for: Marine Community Metabolomes Carry Fingerprints of Phytoplankton Community Composition
Source: mSystems. 2021 May 4;6(3):e01334-20. doi: 10.1128/mSystems.01334-20 (PMC8269262; doi:10.1128/mSystems.01334-20)
Supplement: TABLE S3 [file msystems.01334-20-st003.pdf]

Broad taxon	Species	Strain	*n*	Short ID
Archaea	*Nitrosopumilus maritimus*	SCM1	3	Nmar
Cyanobacteria	*Crocosphaera watsonii*	8501	3	8501
Cyanobacteria	*Prochlorococcus marinus*	1314	3	1314P
Cyanobacteria	*Prochlorococcus marinus*	AS9601	3	As9601
Cyanobacteria	*Prochlorococcus marinus*	MED4	3	MED4
Cyanobacteria	*Prochlorococcus marinus*	NATL2A	3	Nat
Cyanobacteria	*Synechococcus sp.*	7803	2	7803
Cyanobacteria	*Synechococcus sp.*	8102	2	8102
Diatom	*Cyclotella meneghiniana*	338	3	Cy
Diatom	*Navicula pelliculosa*	543	3	Np
Diatom	*Phaeodactylum tricornutum*	2561	2	Pt
Diatom	*Pseudo-nitzschia pungens*	Pc55x	3	Pc55x
Diatom	*Thalassiosira oceanica*	1005	3	To
Diatom	*Thalassiosira pseudonana*	1335	3	Tp
Dinoflagellate	*Alexandrium tamarense*	1771	3	1771
Dinoflagellate	*Amphidinium carterae*	1314	3	1314
Dinoflagellate	*Heterocapsa triquetra*	449	3	449
Dinoflagellate	*Lingulodinium polyedra*	2021	3	2021
Haptophyte	*Emiliania huxleyi*	2090	3	2090
Haptophyte	*Emiliania huxleyi*	371	3	371
Prasinophyte	*Micromonas pusilla*	1545	3	1545
Prasinophyte	*Ostreococcus lucimarinus*	3430	3	3430

TABLE S3 Summary of cultured organisms analyzed in this study. More information (including culturing conditions for all except the Archaea) can be found in (5). Cyanobacteria and archaea were obtained from individual lab culture collections and eukaryotic phytoplankton were obtained from the NCMA culture collection. More detailed information are in Table S7. Short ID is how the organism is labeled throughout the figures.
