# Supplementary figures and images for: Correction for Heal et al., “Marine Community Metabolomes Carry Fingerprints of Phytoplankton Community Composition”
Source: mSystems. 2023 Mar 20;8(2):e01086-22. doi: 10.1128/msystems.01086-22 (PMC10134881; doi:10.1128/msystems.01086-22)

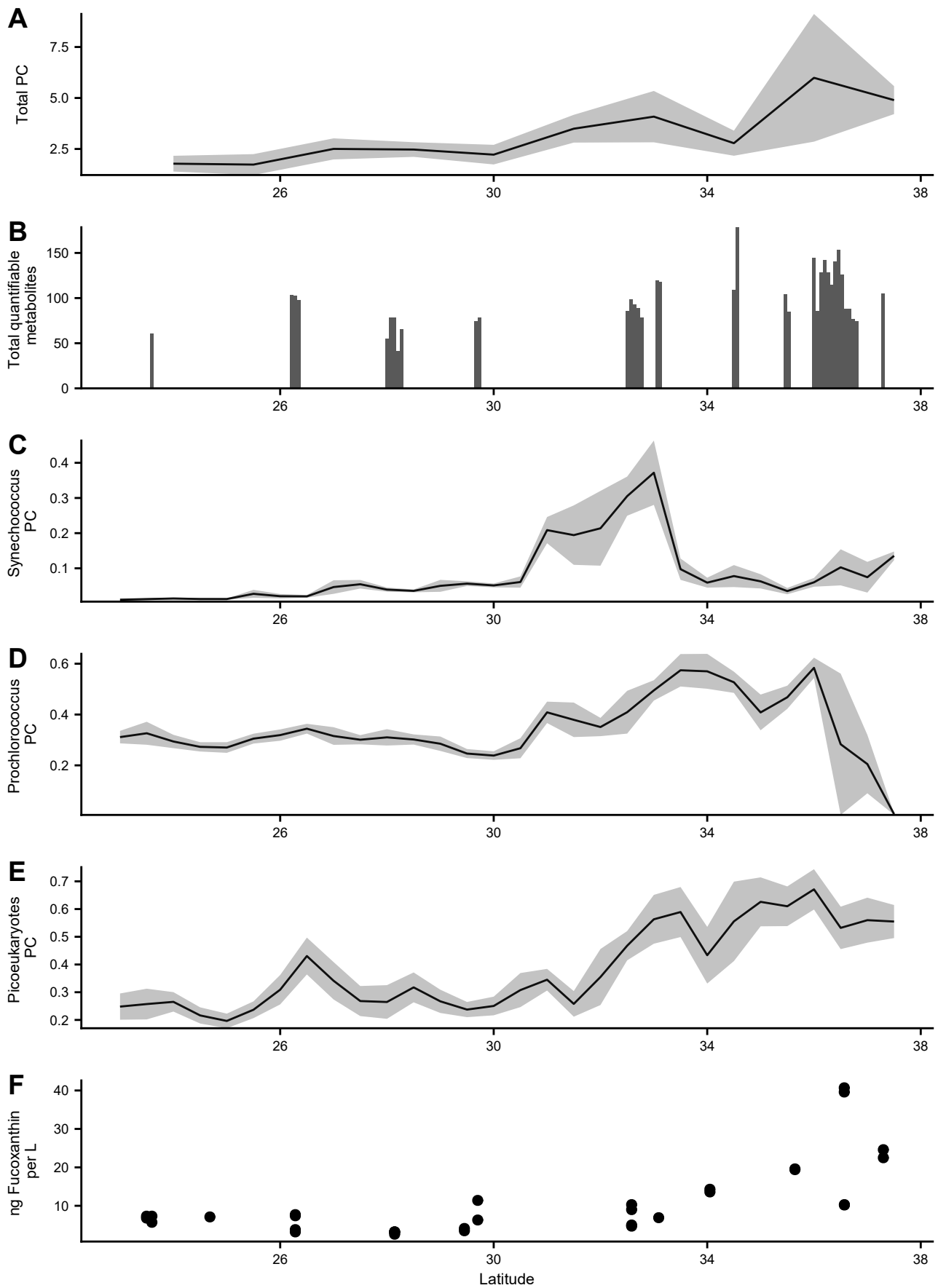

Supplement: FIG S1 [file msystems.01086-22-s0001.pdf]

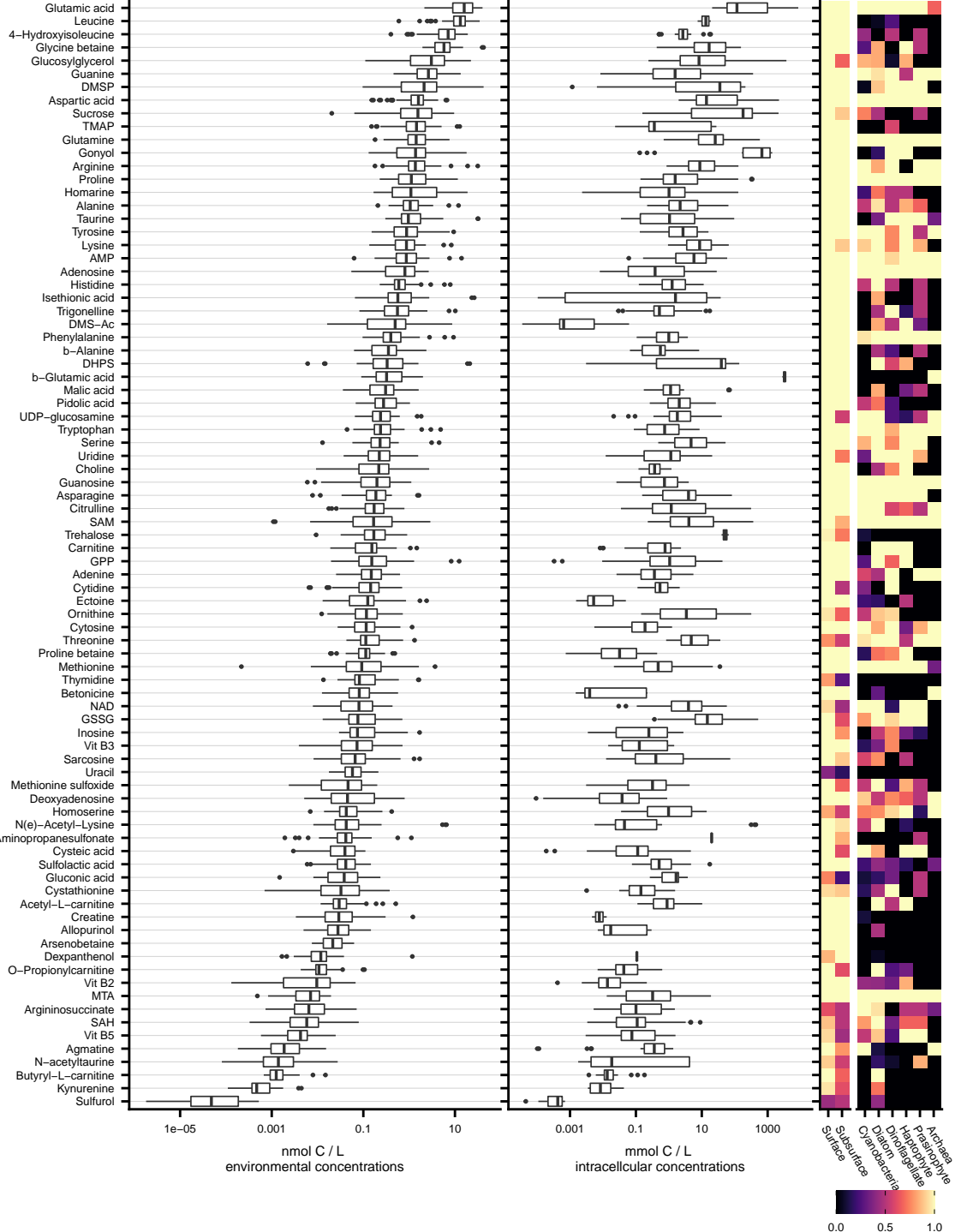

Supplement: FIG S3 [file msystems.01086-22-s0004.pdf]

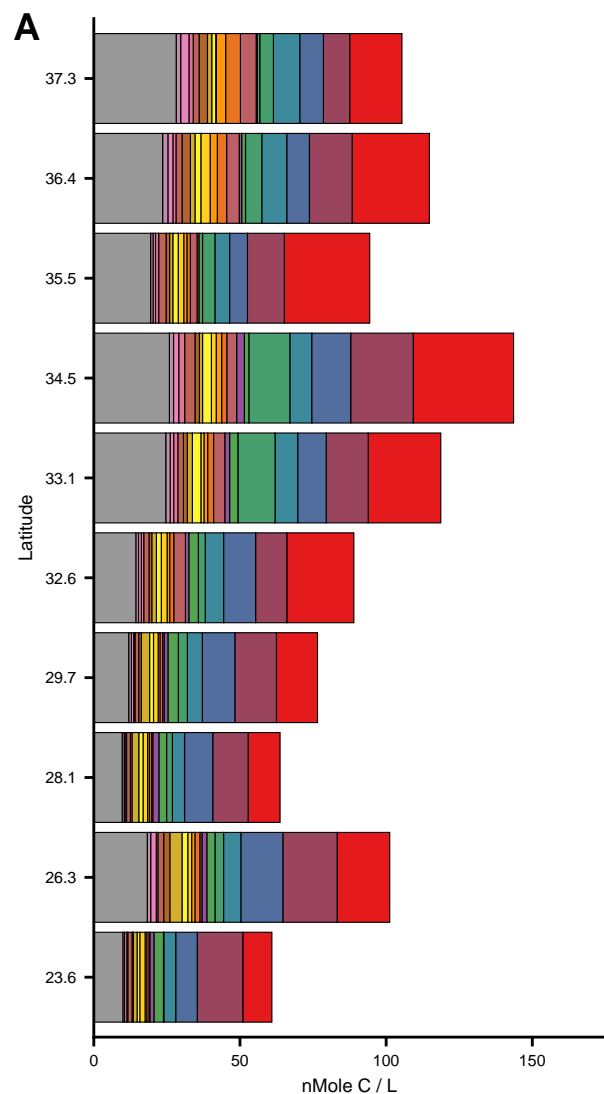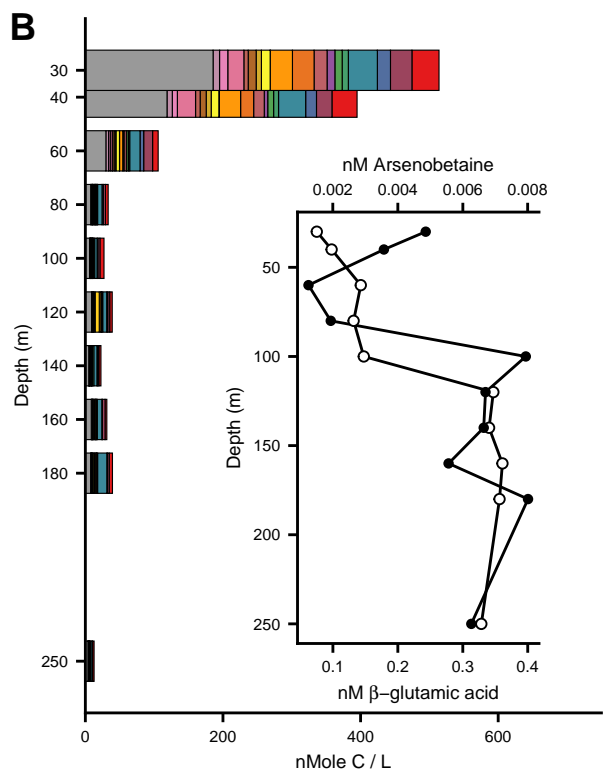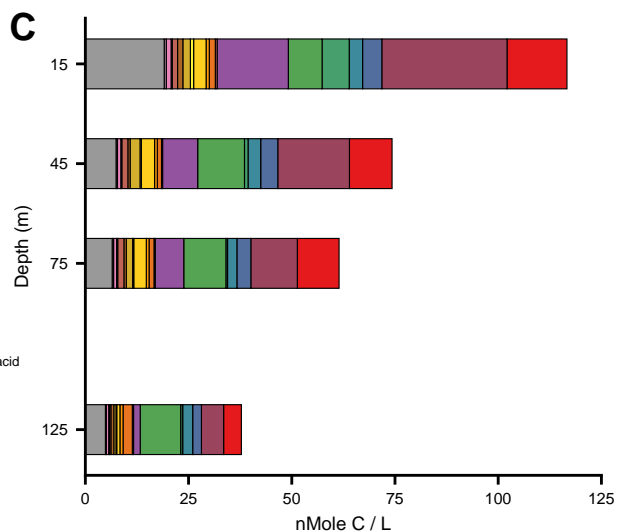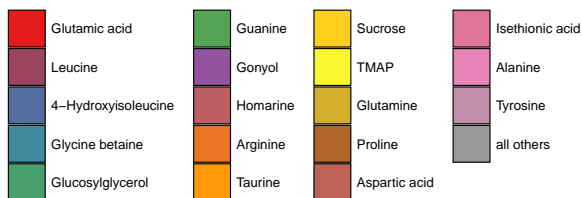

Supplement: FIG S4 [file msystems.01086-22-s0003.pdf]

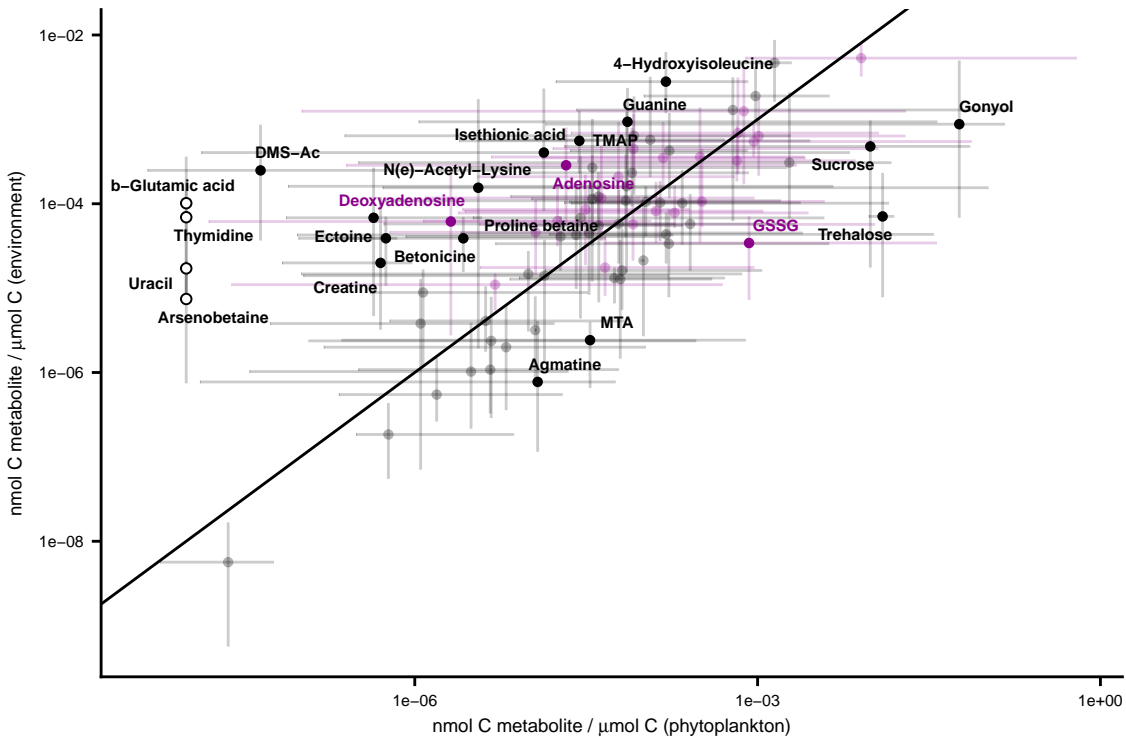

Supplement: FIG S5 [file msystems.01086-22-s0005.pdf]
